# Efficacy of traditional Chinese medicine on diabetic cardiomyopathy in animal models: a systematic review and meta-analysis

**DOI:** 10.3389/fphar.2023.1253572

**Published:** 2023-09-28

**Authors:** Longxiao Hu, Longxin Qian, Aochuan Sun, Guida Cai, Yunxiao Gao, Yue Yuan, Xiaoxiao Chen, Yunyao Jiang, Jianxun Liu, Junguo Ren

**Affiliations:** ^1^ Xiyuan Hospital, Institute of Basic Medical Sciences, China Academy of Chinese Medical Sciences, Beijing, China; ^2^ Beijing University of Chinese Medicine, Beijing, China; ^3^ Institute of Chinese Medicine, Guangdong Pharmaceutical University, Guangzhou, China; ^4^ Institute for Chinese Materia Medica, School of Pharmaceutical Sciences, Tsinghua University, Beijing, China

**Keywords:** diabetic cardiomyopathy, traditional Chinese medicine, animal model, systematic review, meta-analysis

## Abstract

**Background:** Diabetic cardiomyopathy (DCM) is a severe complication of diabetes that can diminish the quality of life in patients and is a leading cause of death. Research has demonstrated the effectiveness of Traditional Chinese Medicine (TCM) in reducing blood sugar levels and protecting cardiovascular function in both animal models and clinical research studies. Nevertheless, the efficacy of TCM in animal models of DCM has not been analyzed systematically.

**Method:** We searched the following electronic bibliographic databases: Web of Science, PubMed, Cochrane Library, and CNKI(China National Knowledge Infrastructure). Studies that reported the efficacy of TCM in animals with DCM were included. The literature search was conducted using the terms. The data will be restricted from the year 2013 to 24 April 2023, 24 studies were included in the meta-analysis.

**Result:** A total of 24 Traditional Chinese Medicine interventions and 2157 animals met the inclusion criteria. The pooled data revealed that TCM interventions resulted in significant improvements in body weight (BW), heart weight (HW) to body weight ratio (HW/BW), triglyceride (TG) and cholesterol (TC) levels, ejection fraction (EF), fractional shortening (FS) and E/A ratio. Subgroup analysis and meta-regression revealed that the type of TCM, duration of intervention, method of modeling, and animal species were potential sources of heterogeneity.

**Conclusion:** TCM interventions were associated with significant improvements in body weight, heart weight to body weight ratio, triglyceride and cholesterol levels, left ventricular internal dimension in systole, ejection fraction, fractional shortening and E/A ratio. The heterogeneity in the results was found to be potentially due to the type of TCM, duration of intervention, method of modeling, and animal species, as shown in subgroup analysis and meta-regression.

**Systematic Review Registration:** identifier CRD42023402908

## 1 Introduction

With the global population aging rapidly and the increasing prevalence of diabetes mellitus, there is a staggering increase in the prevalence of diabetes mellitus-induced cardiomyopathy. This condition is characterized by a higher mortality rate compared to patients without diabetes (Lehrke and Marx, 2017; Nakamura et al., 2022). Cardiovascular diseases are the leading cause of death in this population (Loffroy et al., 2009). Despite significant research advances in diabetes and heart disease, the concept of diabetic cardiomyopathy (DCM) remains controversial and not standardized (Seferovic et al., 2018). Diabetic cardiomyopathy is a cardiac manifestation exclusive to patients with diabetes, which is characterized by left ventricular hypertrophy, diastolic dysfunction, and, in late stages, by significant heart failure and reduced systolic function (Paolillo et al., 2019).

**FIGURE 1:**
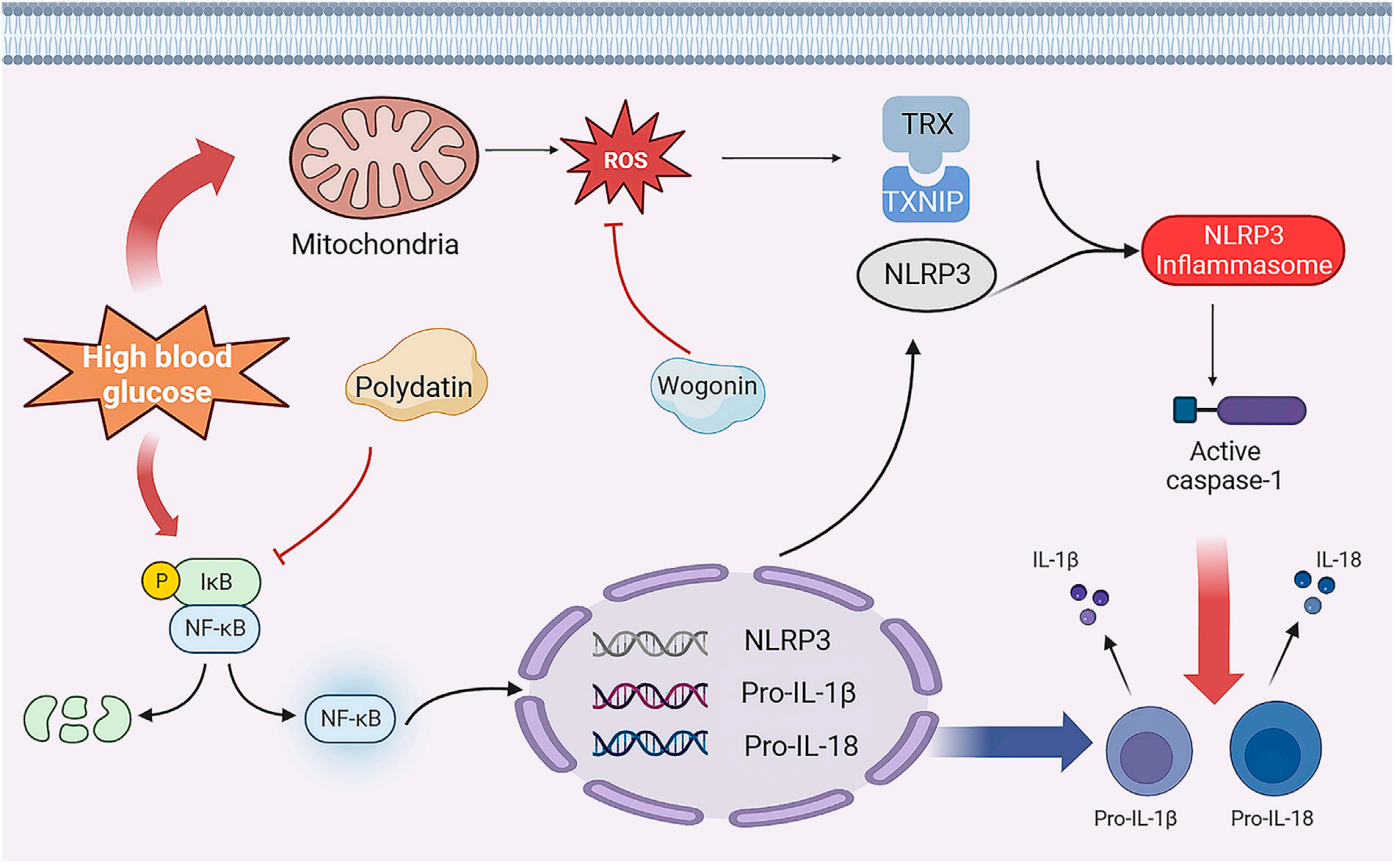
Pathological mechanism of DCM and therapeutic mechanism of TCM

Diabetes mellitus impairs cardiac metabolic flexibility (Jankauskas et al., 2021). Diabetes-related hyperinsulinemia and insulin resistance increase the risk of capillary injuries and can lead to myocardial fibrosis, myocardial hypertrophy, and impaired mitochondrial function. Insulin signaling is reduced in both type 1 diabetes (T1D) and type 2 diabetes (T2D), which is a characteristic feature of these conditions. Additionally, there are changes in other signaling pathways, such as decreased AMPK (AMP-activated protein kinase) signaling and increased PKC (protein kinase C) and MAPK (mitogen-activated protein kinase) signaling. These alterations have detrimental effects on the body’s adaptive response (Dillmann, 2019). Furthermore, excessive lipotoxicity from fat deposits or lipid droplets can affect cardiomyocytes, leading to increased oxidative stress and inflammation, ultimately resulting in cardiac fibrosis and hypertrophy (Nakamura et al., 2022). Elevated glucose or fatty acid levels activate NF-κB(Nuclear factor κB) in the myocardium (Lawrence, 2009), which leads to the formation of NLRP3 (nucleotide-binding domain-like receptor protein 3) inflammatory vesicles that release pro-inflammatory mediators (Shao et al., 2015). High glucose levels also disrupt mitochondrial function, generating ROS(Reactive oxygen species) (He et al., 2021). These ROS cause the aggregation of TXNIP(Thioredoxin-interacting protein) with TRX (Thioredoxin), leading to the activation of the NLRP3 inflammasome. This inflammasome activates caspase-1, which processes pro-inflammatory cytokines IL-1β and IL-18. ROS further stimulate NF-κB, facilitating the assembly of the NLRP3 inflammasome and cleavage of caspase-1 (Zhou et al., 2011; Couchie et al., 2017). This cascade results in the release of IL-1β and IL-18, potent inflammatory molecules in diabetes (Figure 1). Activated endothelial cells in diabetes contribute to early myocardial stiffness and diastolic dysfunction (Franssen et al., 2016). In this condition, these cells disrupt the normal functioning of eNOS(endothelial-type nitric oxide synthase), leading to the generation of deleterious substances like superoxide, hydrogen peroxide, and peroxynitrite ions. This process also contributes to a decrease in the levels of NO(nitric oxide), amplifying the detrimental effects on the myocardium (Evan et al., 2019).

To diagnose DCM, clinicians must exclude various contributing factors, including valvular, coronary, hypertensive, or congenital heart disease, along with familial, viral, toxic, or infiltrative cardiomyopathies. Additionally, they must evaluate for glycometabolic disorders before a definitive diagnosis of DCM can be made (Seferović and Paulus, 2015). Non-coding RNAs have emerged as potential diagnostic criteria in the pathogenesis of diabetic cardiomyopathy in recent years (Guo and Nair, 2017).

Currently, there are no specific treatments for DCM, and clinical treatments for diabetes and cardiomyopathy remain separate (Lorenzo-Almorós et al., 2022). However, treatments for diabetes have varying effects on patients with cardiomyopathy. Nakamura et al. (2022) described diabetic treatments including Caloric Restriction, Sulfonylureas, Insulin, Thiazolidinediones, Dipeptidyl Peptidase-4 (DPP4) Inhibitors, and Sodium-Glucose Cotransporter 2 (SGLT2) Inhibitor. These treatments can have varying cardiovascular effects. Studies have shown that for diabetics, intensive glycemic control decreases the microvascular complications, while macrovascular complications remain unaffected; it is a complex systemic phenomenon of the disease (Eriksson and Nyström, 2015). SGLT2 can be considered one of the most effective treatments for DCM. However, almost all of these treatments are associated with side effects or limitations, and patients with stable blood glucose levels may still experience adverse effects (Xiao and Luo, 2018). For instance, thiazolidinediones (TZDs) and dipeptidyl peptidase-4 (DPP4) can increase the risk of heart failure (Sc et al., 2015).

The form of DCM leads to overt heart failure with an increased death rate remains to be investigated (Ernande and Derumeaux, 2012). Traditional Chinese medicine (TCM) is widely used to treat disease and preserve health, a series of animal experiments have manifested that TCM can improve glucose metabolism (Wang et al., 2020), reduce the inflammatory response (Meng et al., 2022) and inhibit cardiomyocyte remodeling to relieve symptoms of DCM. However, the efficiency of Traditional Chinese Medicine in animals has not been systematically reviewed, which hinders the treatment of DCM. In order to reveal the effectiveness of TCM in treating diabetic cardiomyopathy using animal studies, here, we performed a systematic review and meta-analysis of these studies, as preclinical evidence.

## 2 Methods

We performed this systematic review and meta-analysis in accordance with the Preferred Reporting Items for Systematic Reviews and Meta-Analyses guidelines (Lorenzo-Almorós et al., 2022). The efficacy outcomes assessed include BW(body weight), HW/BW(heart weight/body weight), BG (blood glucose), TG (triglyceride) levels, serum TC (total cholesterol) levels, LVIDs (left ventricular internal dimension in systole), EF (ejection fraction), FS(fractional shortening), and E/A ratio. Decreased body weight, high heart weight/body weight ratio, high blood glucose, increased triglyceride and serum total cholesterol levels, lower ejection fraction and fractional shortening were considered as indicators of diabetic cardiomyopathy.

### 2.1 Search strategy

We conducted this report based on the Preferred Reporting Items for Systematic Reviews and Meta-Analyses checklist. This study was registered with PROSPERO (registration number: CRD42023402908). We searched the following electronic bibliographic databases: Web of Science, PubMed, Cochrane Library, and CNKI(China National Knowledge Infrastructure). Studies that reported the efficacy of TCM in animals with DCM were included. The literature search was conducted using the terms (“Diabetic cardiopathy”) OR (“diabetic cardiomyopathy”)OR (“Autonomic neuropathy") OR (“microangiopathy”) OR (“microvascular disease”) AND (“animal” OR" animals” OR “rat” OR” rats” OR” mouse”OR” mice”) AND (“Chinese medicine “OR” Chinese herb ” OR” herbal ” OR ” natural drug” OR “Natural Product” OR “traditional Chinese” OR “formula”). The date will be restricted from the year 2013 to 24 April 2023. The English and Chinese languages will be searched only. All searches were performed by two independent researchers (LH and LQ).

### 2.2 Inclusion and exclusion criteria

The criteria for inclusion in this study consist of animal models with diabetic cardiomyopathy without limitations regarding the construction, design types, randomized controlled trials, sexes and species. All studies that comprise the number of animals used and TCM treatments, including a single Chinese herbal extract (monomer), compounds of several Chinese herbal extracts, and Chinese herbal compounds, were included. Controlled studies with one or more separate control groups and those published in Chinese or English were also included. On the other hand, the exclusion criteria consisted of cell and *in vitro* studies, studies that used other non-TCM treatments, case reports, clinical trials, reviews, and cross-over studies that lacked a separate control group. Furthermore, studies that did not provide data were not included. Two researchers (AS and JR) independently screened the studies, and discrepancies were resolved through discussions.

### 2.3 Data extraction

Data extraction from the included studies was conducted by two independent researchers (LH and LQ). The following data were extracted from the studies: outcome measures (body weight, HW/BW, blood glucose, triglyceride, serum total cholesterol, left ventricular end-systolic and end-diastolic diameter, ejection fraction, fractional shortening, and E/A ratio), publication information (author and year), details of treatments administered (dose, route, and duration), and the species of animals used. The study extracted sample size, mean value, and standard deviation (SD)/standard error for the both control and treatment groups for each comparison. In cases where multiple treatment groups shared the same control group, the sample size of the control group was divided by the number of treatment groups for appropriate comparison (Eriksson and Nyström, 2015). If outcomes were measured at more than one time point, we only included data from the last time point (Xiao and Luo, 2018).

### 2.4 Risk of bias assessment

The included studies were independently assessed for quality by two researchers, and disagreements were resolved through consultation with a third party. The quality of the included studies was evaluated using the SYRCLE (Systematic Review Center for Laboratory Animal Experimentation) Risk of Bias tool. The assessment includes 11 areas that are scored and evaluated separately. These areas are as follows: 1) Random sequence generation; 2) Baseline characteristics; 3) Allocation concealment; 4) Random housing; 5) Blinding; 6) Blinding of participants and personnel; 7) Random outcome assessment; 8) Blinding of outcome assessment; 9) Incomplete outcome data; 10) Selective reporting; 11) Other bias. Any discrepancies that arose during the evaluation were resolved through discussion until a consensus was reached.

### 2.5 Data analysis

Data were synthesized using either a fixed effects model or a random effects model based on a heterogeneity test. Heterogeneity between studies was explored by Cochran’s Q statistic and I^2^statistic (Diao et al., 2019). All data were analyzed using Review Manager version 5.4 and STATA version 17 packages. Standardized mean differences (SMD) were used as an index of effect for each effect, their point estimates, and 95% confidence intervals. Heterogeneity between included studies was analyzed using the χ^2^ test, while the magnitude of heterogeneity was determined quantitatively using I^2^. If heterogeneity between results was not significant (I^2^ ≤ 50%), meta-analysis was performed using a fixed effects model (EF, FS, and E/A). If the heterogeneity between studies was significant (I^2^>50%), a random effects model was used (body weight, HW/BW, BG, TG, TC, and LVIDs). The study’s findings were mainly presented using forest plots, with funnel plots utilized to analyze publication bias. Additionally, sensitivity analyses and Egger’s test were carried out (Feidantsis et al., 2018). To further explore the sources of heterogeneity, subgroup analyses should be conducted on the types of Traditional Chinese Medicine (TCM), duration, modeling method, and animal species.

## 3 Results

### 3.1 Characteristics of included studies

**FIGURE 2:**
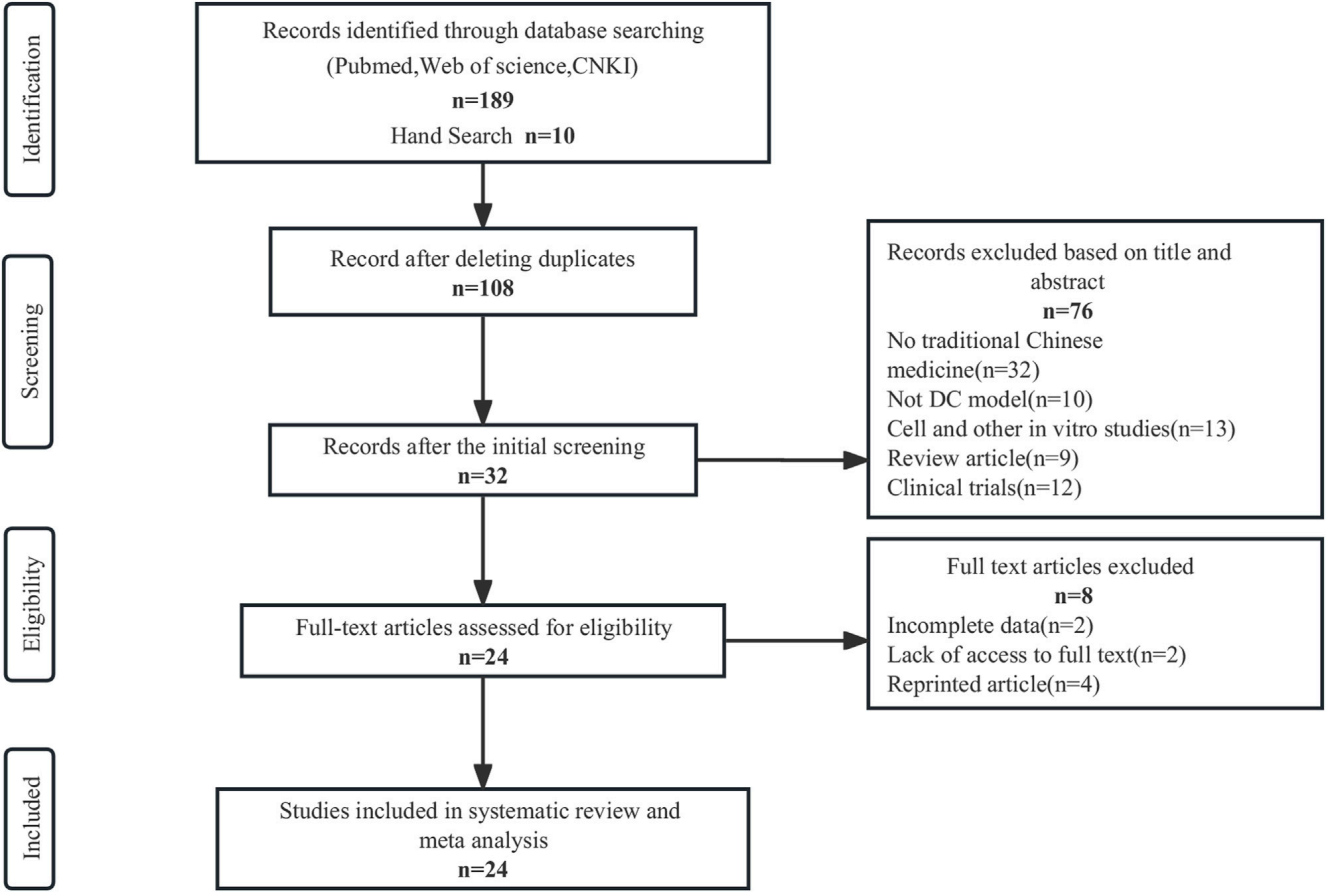
Flow diagram of the study selection process.

In this study, a search of 199 publications was conducted, with 168 articles obtained from PubMed, 19 from CNKI, 2 from Web of Science, and 10 from manual search. The duplicates were screened using Endnote 20, resulting in 91 exclusions. Based on the title and abstract assessment, 76 articles were excluded manually, and 8 out of the remaining 32 articles were excluded after reading the full text for not meeting the inclusion criteria. Eventually, a total of 24 articles were included in this study. Please refer to Figure 2 for the screening process details (Page et al., 2021). Altogether 2157 animals (1088 in the TCM group; 1069 in the control group) were included in this meta-analysis. Ten studies used Sprague Dawley rats, seven studies used Wistar rats, six studies used C57BL/6 J mice, and one study used Kunming mice. Rats weighed between 70–280 g, while C57BL/6 J mice and Kunming mice weighed between 23-27.5 and 18–22 g, respectively. The age of the rats was only reported in ten studies and ranged from 6 to 12 weeks. The studies used three different models: High-fat food (HFD) and streptozotocin (STZ) in eleven studies, STZ alone in ten studies, and alloxan in three studies.

Regarding the intervention methods, the researchers identified 24 different types of TCM, including ten compounds and fourteen monomers. The monomers consisted of Berberine (An isoquinoline alkaloid, mainly derived from Rhizoma Coptidis [Ranunculaceae; Coptis chinensis Franch. rhizome]), Curcumin (A yellow curry pigment isolated from Turmeric [Zingiberaceae; Curcuma longa L. root]), Crocin (An extract of Crocus sativus [Iridaceae; Crocus sativus L. flower]), Dioscin (An extract of Dioscoreaceae [Dioscoreaceae; Dioscorea oppositifolia L. rhizome]), Mangiferin (A carboketone glycoside of tetrahydroxypyridone from rhizoma anemarrhenae [Asparagaceae; Anemarrhena asphodeloides Bunge root]), Resveratrol (A non-flavonoid polyphenolic organic compound isolated from the roots of Veratrum grandiflorum [Melanthiaceae; Veratrum grandiflorum root]), Myricetin (A flavonol compound extracted from the bark of Myrica rubra [Myricaceae; Myrica rubra]), Nar (A dihydroflavonoid from pomelo [Rutaceae; Citrus paradisi Macfadyen L. fruit]), Polydatin (An extract of Plantago Ovata [Plantaginaceae; Plantago ovata Forssk. rhizome]), Rutin (A natural flavonoid glycoside present in Sophora japonica rice [Fabaceae; Styphnolobium japonicum L. buds]), Sal B (the representative component of phenolic acids derived from the Salvia miltiorrhiza Bunge [Lamiaceae; Salviae miltiorrhizae radix et rhizoma]), taxifolin (a flavonoid commonly found in Pseudotsuga taxifolia [Pinaceae; Pseudotsuga menziesii var. menziesii]), triptolide (a most important active ingredient extracted from Tripterygium [Celastraceae, Tripterygium wilfordii Hook.f.]), and Wogonin (an important flavonoid derived from the Scutellaria [Lamiaceae; Scutellaria baicalensis Georgi root]). The intervention durations ranged from 24 to 180 days. Intraperitoneal administration was performed in two of the 24 studies, while gastrointestinal administration was used in the remaining 22 studies. The primary outcome measurements included BW (reported in 11 studies), HW/BW (reported in 7 studies), BG (reported in 15 studies), TG (reported in 7 studies), TC (reported in 10 studies), LVIDs (reported in 6 studies), EF (reported in 8 studies), FS (reported in 8 studies), and E/A (reported in 4 studies). The detailed characteristics of the included studies are shown in Table 1.

**TABLE 1:** Characteristics of included studies.

References	TCM drug	Route	Duration (days)	Species	Weight(g)	Age (weeks)	Model	N (T/C)	Treat	Control	Possible mechanisms	Outcome measures
Chang et al. (2015)	Berberine	i.g	112	Wistar rats	NR	6	HFD + STZ	(8/8)	100 mg/kg/d	Vehicle	The activation of AMPK and AKT was increased, while the activation of GSK3β was reduced	BG, TG, TC
Guo et al. (2016)	triptolide	i.g	56	SD rats	160–170	6	HFD + STZ	(10/10)	50 μg/kg/d 100 μg/kg/d 200 μg/kg/d	Vehicle	inhibition of TLR4-induced NF-κB/IL-1β immune pathway, suppression of NF-κB/TNF-α/VCAM-1 mediated inflammatory pathway and downregulation of TGF-β1/α-SMA/Vimentin involved cardiac fibrosis	BG, HW/BW, TC
Hou et al. (2016)	Mangiferin	i.g	112	SD rats	220–240	NR	HFD + STZ	(8/8)	20 mg/kg/d	Vehicle	inhibition of ROS overproduction, AGEs/RAGE, and inflammatory activation via deactivating of NF-κB translocation	BG, TG, TC
Li et al. (2020)	Sal B	i.p	112	C57BL/6 J mice	NR	8	STZ	(20/20)	15 mg/kg/d 30 mg/kg/d	Vehicle	promoting the effect of nuclear translocation and DNA methylation of IGFBP3 to raise VEGFA and its receptor VEGFR2	EF, FS, E/A
Shen et al. (2014)	SSYX	i.g	28	Wistar rats	180–220	NR	HFD + STZ	(5/5)	50 mg/kg/d 100 mg/kg/d 200 mg/kg/d	NR	TGF-β1/Smad signaling pathway is involved in diabetic cardiomyopathy and SSYX treatment improves impaired heart function and inhibits fibrosis in diabetes	TC, TG, HW/BW, LVIDs
Shi et al. (2019)	DJC	i.g	56	SD rats	160–200	NR	HFD + STZ	(15/15)	270 mg/kg/d 540 mg/kg/d 1080 mg/kg/d	Water	inhibiting Overexpression of TLR4/MyD88/NF- κB signaling pathway proteins that protects myocardial cells from injury induced by high glucose	BG, TC, HW/BW
Sun et al. (2014)	taxifolin	i.g	28	C57BL/6 mice	NR	(6–8)	STZ	(10/10)	25 mg/kg/d 50 mg/kg/d 100 mg/kg/d	Vehicle	decreasing angiotensin II production, inhibiting NADPH oxidase, and activating JAK2/STAT3 cascade	BW
Tan et al. (2020b)	polydatin	i.g	56	SD rats	200–220	NR	HFD + STZ	(7/7)	85.71 mg/kg/d	Vehicle	mediating through its inhibition of NADPH oxidase and NF-κB activation	E/A, EF, FS
Wang et al. (2021)	FTZ	i.g	30	C57BL/6 mice	NR	(8–12)	STZ	(4/4)	3 g (crude drug)/kg/d	Vehicle	1) inhibited the activities of AKT1, ERK, and STAT3	BW, BG, EF, FS, E/A
											(2) decreased the interventricular septal thickness	
Wang et al. (2018)	YQHX	i.g	24	SD rats	200–220	NR	HFD + STZ	(6/6)	7.5 mL/kg/d	Vehicle	enhancing the expression of the Bcl-2 protein, inhibiting the expression of Bax and P53, increasing the ratio of Bcl-2 and Bax, and inhibiting the apoptosis of cardiomyocytes	BW, BG, HW/BW
Wei et al. (2019)	Dioscin	i.g	42	SD rats	225–250	8	STZ	(10/10)	100 μg/kg/d 200 μg/kg/d	Saline	modulating the NO-sGC-cGMP-PKG signaling pathway, resulting in improved left ventricle function	BW, HW/BW
Wu et al. (2021)	QLQX	i.g	56	SD rats	NR	NR	STZ	(6/6)	0.5 g/kg/d	Saline	inhibited hyperglycemia-induced cardiomyocyte apoptosis in NRCMs via activating PPARγ	LVIDs, E/A
Yao et al. (2021)	THJ	i.g	84	C57BL/6 mice	23–25	(8–10)	HFD + STZ	(15/15)	0.125 g/kg/d 0.25 g/kg/d 0.5 g/kg/d	Vehicle	reducing ROS production and inhibiting NLRP3 activation, thereby blocking the excessive secretion of pro-inflammatory cytokines	BG, TG
Zhang et al. (2018)	Nar	i.g	56	SD rats	230–280	NR	STZ	(8/8)	25 mg/kg/d 50 mg/kg/d 100 mg/kg/d	Saline	reducing the accumulation of unfolded or misfolded proteins in ER by increasing antioxidant enzyme activity and reducing lipid peroxide production, inhibiting ERS, and reducing the expression of apoptosis-related factors	BW, BG
Zhang et al. (2016)	DOE	i.g	56	Kunming mice	18–22	(8–10)	STZ	(8/8)	75 mg/kg/d 150 mg/kg/d 300 mg/kg/d	Saline	inhibition of oxidative stress, cardiac fibrosis, and downregulation of pro-inflammatory cytokines	BW, HW/BW, BG, TC, TG
Dong et al. (2021)	QGYY	i.g	84	SD rats	180–200	NR	HFD + STZ	(5/5)	5.40 mg/kg/d	Vehicle	improving insulin resistance, inhibiting oxidative stress and inflammatory reaction	BW, BG, TG, TC, LVIDs
Liao et al. (2017)	Myricetin	i.g	180	C57BL/6 Mice	23.5–27.5	(8–10)	STZ	(16/12)	200 mg/kg/d	Saline	inhibiting IκB-α/NF-κB/p65 and TGFβ/Smad signaling and enhancing the expression of Nrf2, which accordingly alleviate oxidative stress, inflammation, apoptosis, and fibrosis	BW, BG, LVIDs, EF, FS
Khan et al. (2016)	Wogonin	i.p	112	C57BL/6 mice	NR	8	STZ	(16/18)	10 mg/kg/3d	Vehicle	suppressing diabetic-induced cardiomyocyte inflammation and oxidative stress	BG, LVIDs, EF, FS
Yu et al. (2012)	Curcumin	Orally	112	Wistar rats	70–90	NR	HFD + STZ	(9/9)	100 mg/kg/d 200 mg/kg/d	Vehicle	activating pro-survival signaling pathway Akt/GSK3β in diabetic hearts. Akt promotes cell survival by inhibiting several targets involved in apoptotic signaling cascades	BW, BG, HW/BW, TC, EF, FS
Diao et al. (2019)	resveratrol	i.g	56	SD rats	100–150	NR	HFD + STZ	(10/10)	10 mg/kg/d	Saline	1) inhibit myocardial fibrosis and apoptosis, and contribute to the protection of the cardiac function in DCM rats	EF, FS
											(2) RES promotes mitochondrial biosynthesis and inhibits excessive mitochondrial division	
Feidantsis et al. (2018)	crocin	Orally	14	Wistar rats	200	NR	STZ	(4/4)	10 mg/kg/d 20 mg/kg/d	Vehicle	1) enhancement of cell protection through upregulation of heat shock response	BW, LVIDs, EF, FS
											2) inhibition of diabetes-induced apoptosis and	
											3) normalization of cardiac autophagy through activation of AMPK	
Ganesan et al. (2020)	Rutin	i.g	30	Wistar rats	180–220	NR	alloxan	(6/6)	100 mg/kg/d	Saline	reducing the over-expression of the aquaporin gene preventing the development of metabolic acidosis	TC
Rahimi-Madiseh et al. (2017)	Allium ampeloprasum (AA)	i.g	56	Wistar rats	200–250	NR	alloxan	(12/12)	400 mg/kg/d 800 mg/kg/d	Water	prevent diabetes and associated complications by decreasing the levels of serum lipids, and significantly increasing the activity of antioxidant enzymes, and decreasing serum MDA	BG, TG, TC
Asgary et al. (2014)	cornelian cherry	i.g	28	Wistar rats	190–220	NR	alloxan	(7/7)	2 g/d	NR	Inhibition of α-glucosidase	BG

Sal B, salvianolic acid B; SSYX, shensong yangxin; DJC, danzhi jiangtang; FTZ, fufang zhenzhutiaozhi; YQHX, yiqi huoxue; QLQX, qiliqiangxin; THJ, taohuajing, Nar, naringin; DOE, debdrobium officinale; QGYY, qiguiyaoye; i. g., intragastric injection; i. p., intraperitoneal injection; NR, not reported; HFD, high fat diet; STZ, streptozotocin; AMPK, Adenosine 5‘-monophosphate (AMP)-activated protein kinase; AKT, protein kinase B; GSK3β, Glycogen synthase kinase 3β; NF-κB, Nuclear factor kappa-B; IL-1β, Interleukin-1β; TNF-α, tumor necrosis factor-α; VCAM-1, vascular cell adhesion molecule-1; TGF-β1, Transforming Growth Factor-β1; α-SMA, α-smooth muscle actin; ROS, reactive oxygen species; AGEs, Advanced Glycation End Products; RAGE, advanced glycosylation end product-specific receptor; IGFBP3, Insulin like growth factor binding protein-3; VEGFA, vascular endothelial growth factor A; VEGFR2, vascular endothelial growth factor receptor 2; TGF-β1, transforming growth factor-β-1; TLR4, Toll-like receptor 4; MyD88, Myeloid differentiation factor 88; NADPH, nicotinamide adenine dinucleotide phosphate; JAK2, Janus kinase 2; ERK, extracellular signal-regulated kinase; STAT3, Signal Transducer and Activator of Transcription 3; Bcl-2, Bcelllymphoma2; Bax, BCL2-Associated X; P53, Cellular tumor antigen p53; NO-sGC-cGMP-PKG, nitric oxide-cyclic guanosine monophosphate)-protein kinase G; NRCMs, neonatal rat cardiomyocyte; PPARγ, peroxisome proliferator-activated receptor γ; NLRP3,NOD-like receptor protein 3; IκB-α, inhibitor kappa B alpha; Nrf2, nuclear factor erythroid-2-related factor 2; RES, reticuloendothelial system; MDA, malondialdehyde; BW, body weight; BG, blood glucose; TG, triglyceride; TC, total cholesterol; LVIDs, left ventricular end systolic diameter; EF, ejection fraction; FS, fractional shortening.

### 3.2 Risk of bias and quality of the included studies

**FIGURE 3:**
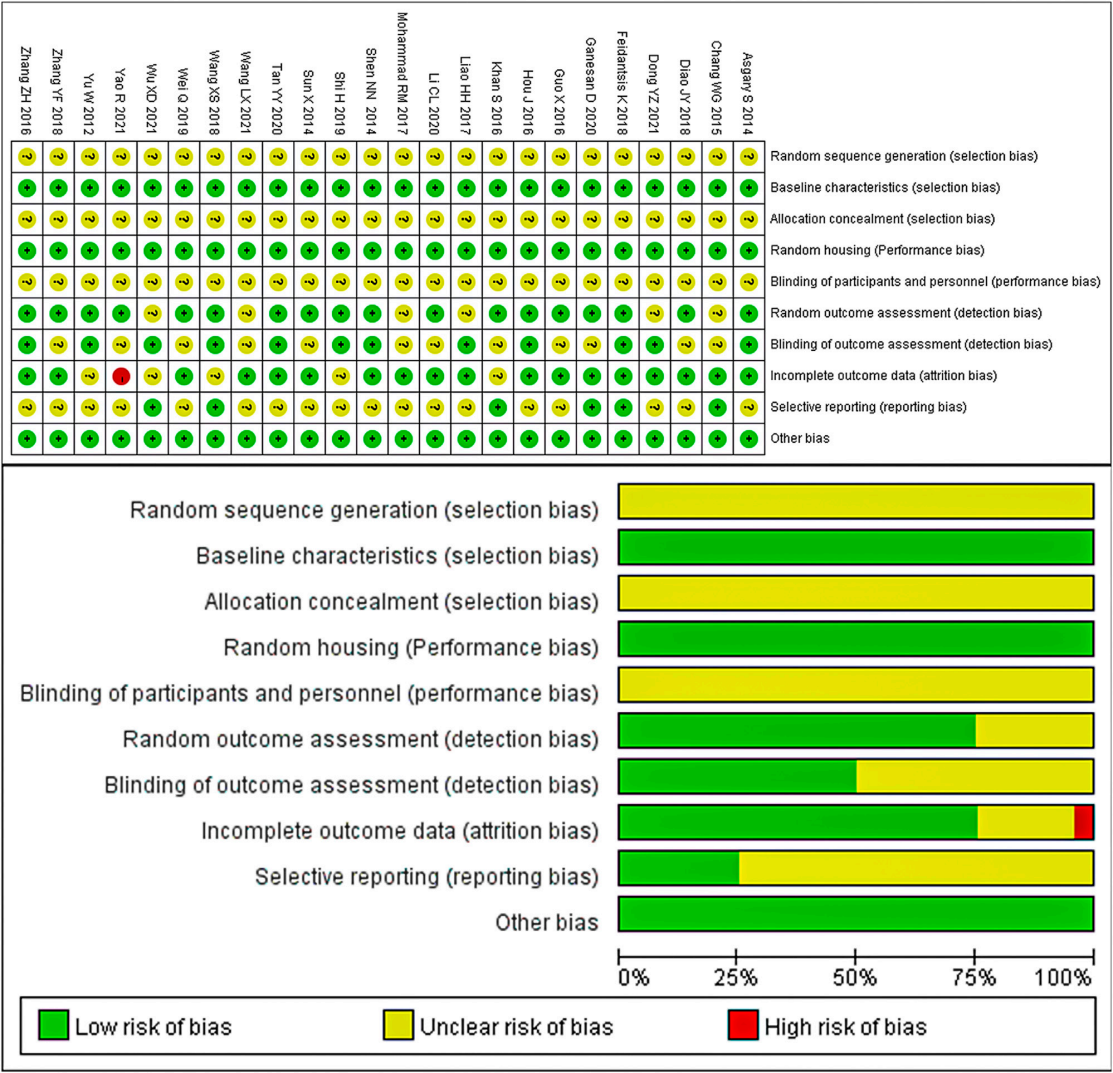
Risk of bias graph and bias summary..

The results of the quality assessment of the 24 included studies are presented in Figure 3. None of the studies reported the random sequence generation, allocation concealment, and blinding of participants and personnel. However, all the studies reported similar baseline characteristics between the model and control groups. Additionally, all experimental animals were housed in identical conditions, so the placement of animals in all studies can be considered consistent with the principle of randomization. One study had a high risk of bias for incomplete outcome data (attrition bias) due to an unclear number of animals. Eighteen studies reported the random outcome assessment, while 12 studies assessed the blinded methods used for outcome assessment. None of the studies had a selective reporting bias or any other sources of bias. Despite the overall poor quality of these studies, none of them were excluded based on quality or risk of bias assessment.

### 3.3 Meta-analysis of the efficacy of TCM

#### 3.3.1 Efficacy of TCM on body weight

**FIGURE 4:**
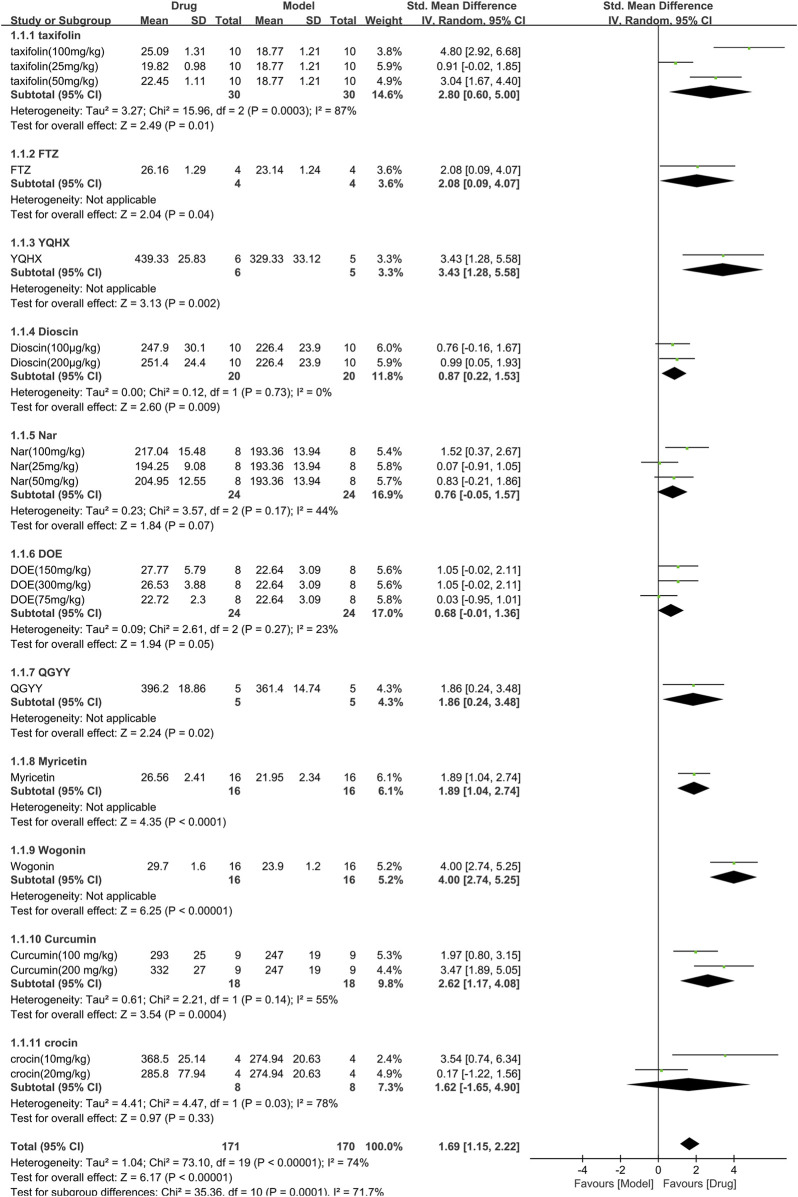
Effects of TCM on body weight(g). Forest plots of the effect size were calculated using SMDs. The horizontal error bars represent the 95% confidence interval of individual studies.

Eleven studies (341 animals, with 171 in the TCM group and 170 in the control group) assessed body weight and were included in the meta-analysis (Figure 4). The pooled results showed that TCM significantly increased body weight [SMD = 1.69 (1.15,2.22), *p* < 0.00001; 20 comparisons], despite significant heterogeneity between studies (χ^2^ = 73.10; I^2^ = 74%; df = 19; *p* < 0.00001). Subgroup analysis indicated a significant correlation (*p* < 0.001). Among the TCM interventions, Nar, DOE, and crocin had similar efficacy to the control group (*p* = 0.07, *p* = 0.05, and *p* = 0.33, respectively), while the other TCM interventions were associated with significant weight gain (*p* < 0.05). The forest plot showed that wogonin had a more prominent effect.

#### 3.3.2 Efficacy of TCM on HW/BW

**FIGURE 5:**
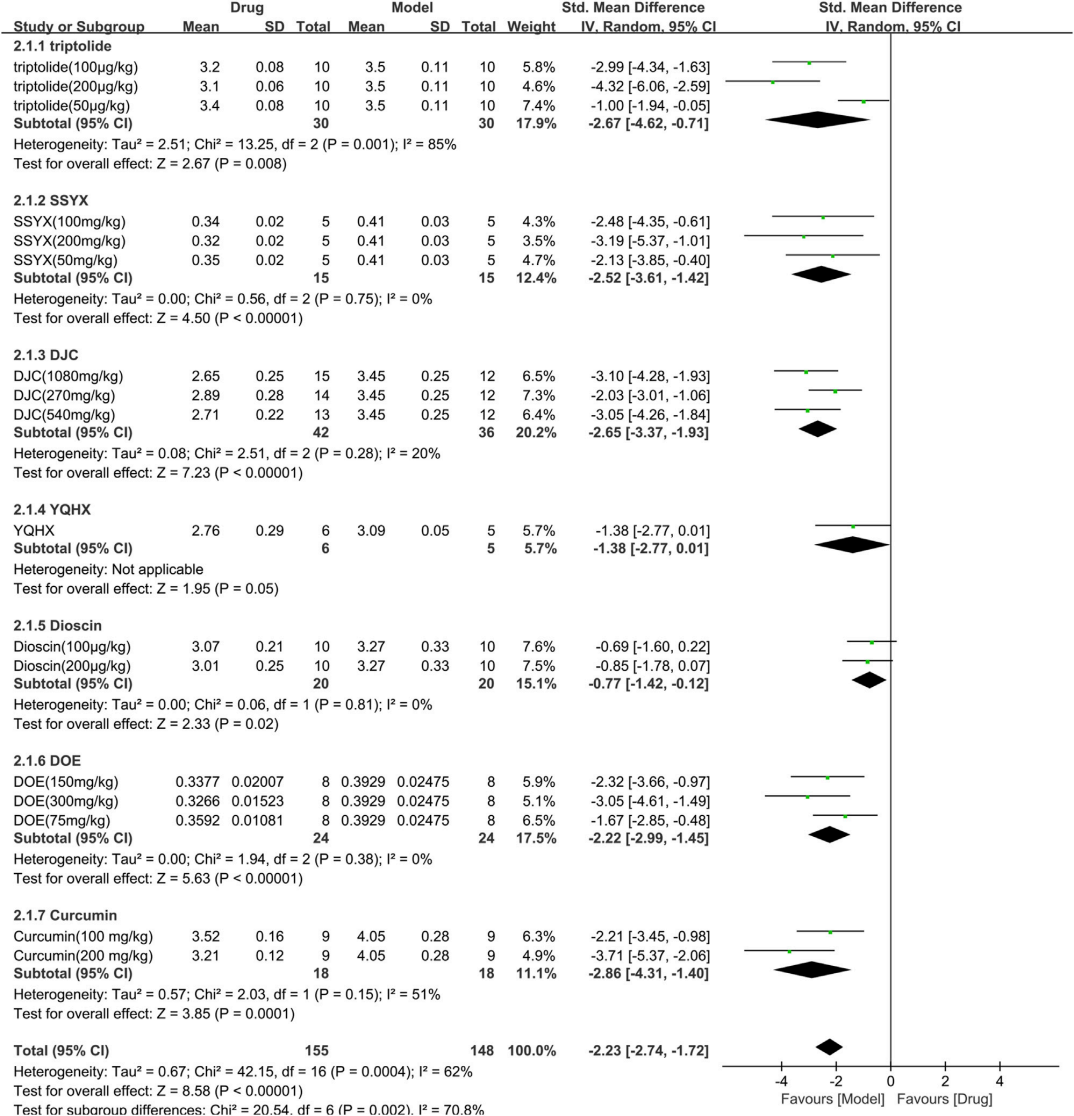
Effects of TCM on HW/BW(mg/g). Forest plots of the effect size were calculated using SMDs. The horizontal error bars represent the 95% confidence interval of individual studies.

The HW/BW was assessed in 7 studies (303 animals, 155 were in the TCM group and 148 were in the control group), which are included in the meta-analysis (Figure 5). The overall analysis demonstrated a significant reduction in HW/BW with the use of TCM [SMD = −2.23 (−2.74, −1.72), *p* < 0.00001; 17 comparisons), but with noticeable heterogeneity across trials (χ^2^ = 42.15; I^2^ = 62%; df = 16; *p* = 0.0004). Subgroup analysis displayed a significant correlation (*p* < 0.01). The efficacy of YQHX was similar to the control group (*p* = 0.05), while the remaining TCM yielded more obvious results than the control group (*p* < 0.05).

#### 3.3.3 Efficacy of TCM on blood glucose

**FIGURE 6:**
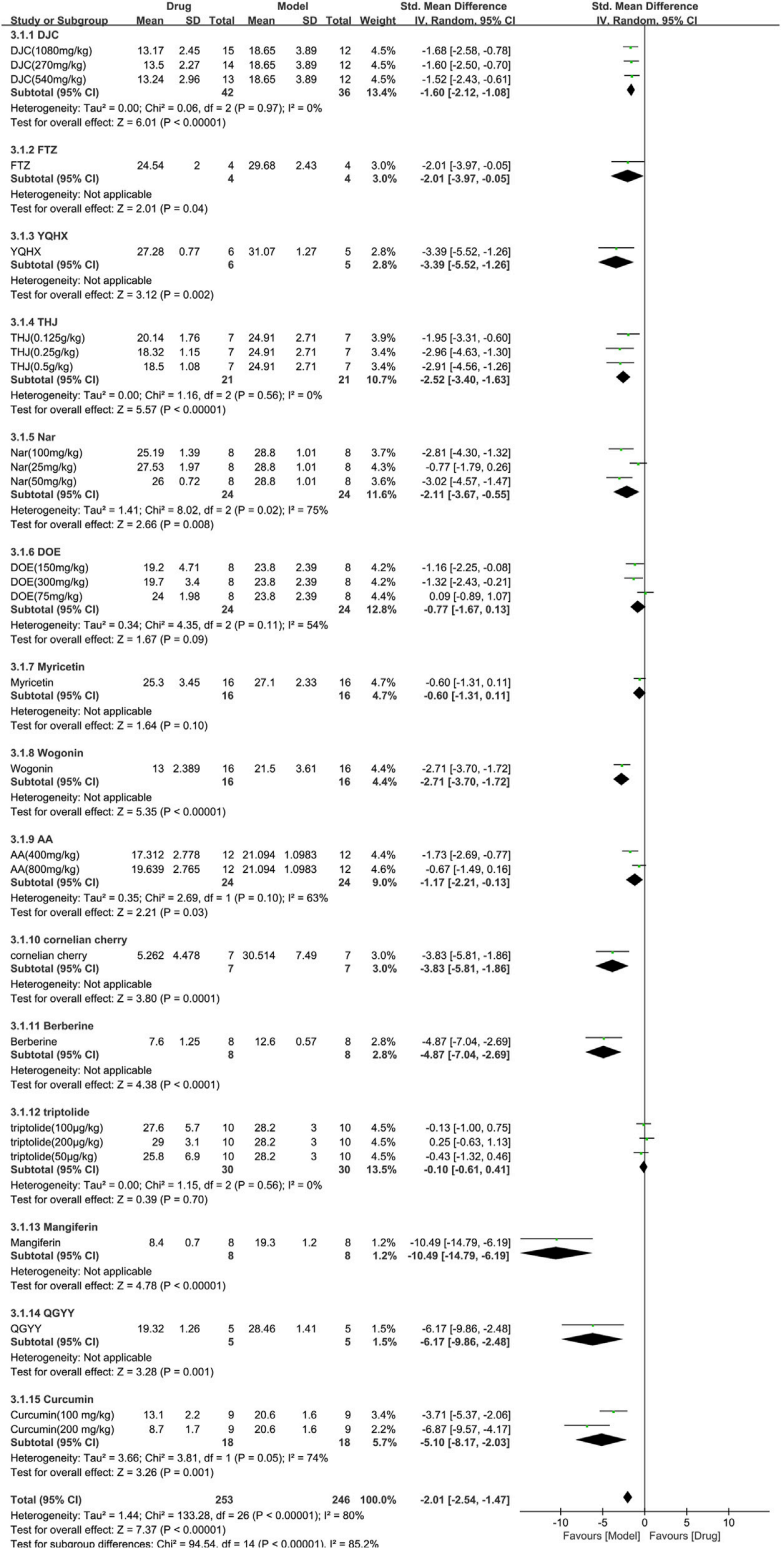
Effects of TCM on blood glucose (mmol/L). Forest plots of the effect size were calculated using SMDs. The horizontal error bars represent the 95% confidence interval of individual studies.

A total of 15 studies with 499 animals (253 in the TCM and 246 in the control group) assessed the blood glucose levels, which were incorporated in the meta-analysis (Figure 6). The findings showed that TCM group had significantly lower blood glucose levels compared to the control group [SMD = −2.01 (−2.54, −1.47), *p* < 0.00001; 27 comparisons) with a high degree of heterogeneity across studies (χ^2^ = 133.28; I^2^ = 80%; df = 26; *p* < 0.00001). Subgroup analysis indicated a significant correlation between TCM type and effect size (*p* < 0.00001). Despite DOE, Myricetin, and triptolide having similar efficacy to the control group (*p* = 0.09, *p* = 0.10, and *p* = 0.70, respectively), the remaining TCM had a more pronounced effect on blood glucose reduction than the control group (*p* < 0.05). The forest map demonstrated mangiferin to be the most effective TCM.

#### 3.3.4 Efficacy of TCM on triglyceride

**FIGURE 7:**
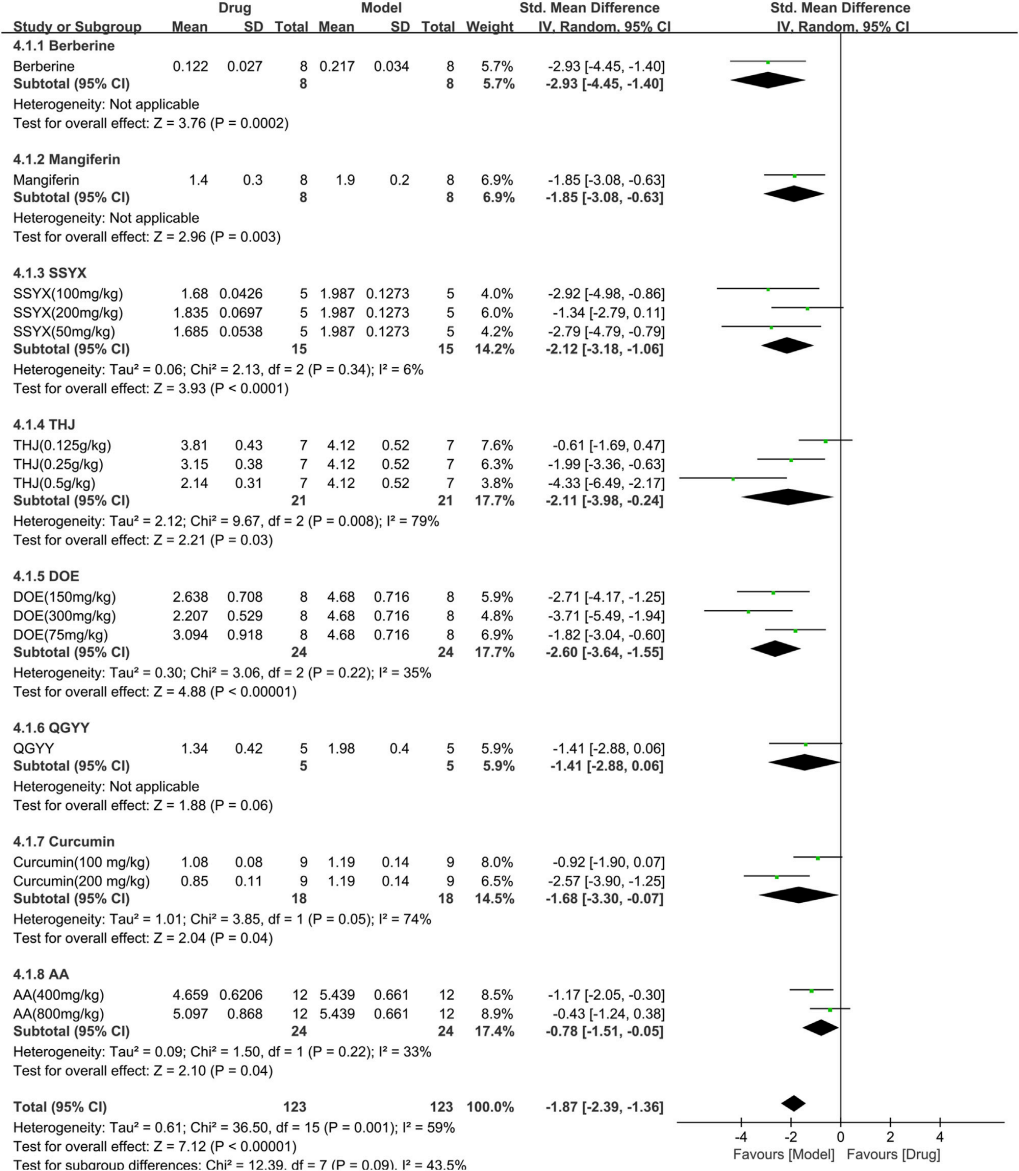
Effects of TCM on triglyceride (mmol/L). Forest plots of the effect size were calculated using SMDs. The horizontal error bars represent the 95% confidence interval of individual studies.

Eight studies (246 animals; 123 in the TCM group and 123 in the control group) analyzed triglyceride levels, which were included in the meta-analysis (Figure 7). The findings demonstrated that TCM significantly reduced triglyceride levels [SMD = −1.87 (−2.39, −1.36),*p* < 0.00001; 16 comparisons) but with noticeable heterogeneity across the studies (χ^2^ = 36.50; I^2^ = 59%; df = 15; *p* = 0.001). Subgroup analysis did not reveal any effect of the TCM type on the results (*p* = 0.09). While QGYY had similar efficacy to the control group (*p* = 0.06), the other TCM yielded a more evident reduction in TG than the control group (*p* < 0.05). The forest map found berberine to have a more marked effect.

#### 3.3.5 Efficacy of TCM on total cholesterol

**FIGURE 8:**
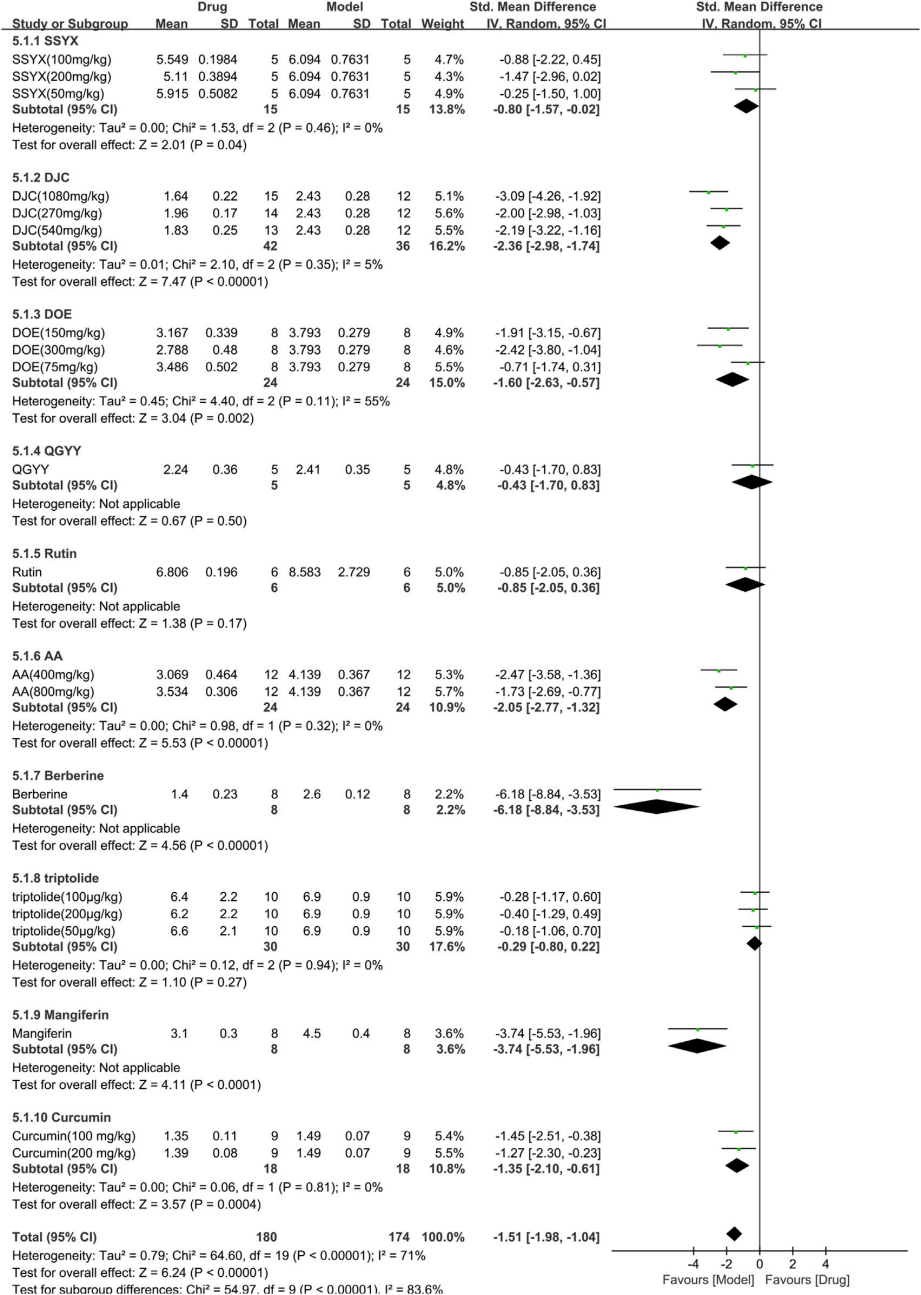
Effects of TCM on total cholesterol (mmol/L). Forest plots of the effect size were calculated using SMDs. The horizontal error bars represent the 95% confidence interval of individual studies.

Ten studies (354 animals; 180 in the TCM group and 174 in the control group) evaluated total cholesterol for inclusion in the meta-analysis (Figure 8). The combined results demonstrated a significant reduction in total cholesterol levels with TCM usage [SMD = −1.51 (−1.98,−1.04), *p* < 0.00001; 20 comparisons) but with marked heterogeneity between studies (χ^2^ = 64.60; I^2^ = 71%; df = 19; *p* < 0.00001). Subgroup analysis indicated a significant correlation (*p* < 0.00001). Whereas QGYY and Rutin showed similar efficacy to the control group (*p* = 0.50, *p* = 0.17, respectively), the other TCM had a more pronounced effect on reducing TC levels than the control group (*p* < 0.05). The forest map revealed that DJC was more effective.

#### 3.3.6 Efficacy of TCM on left ventricular internal dimension in systole

**FIGURE 9:**
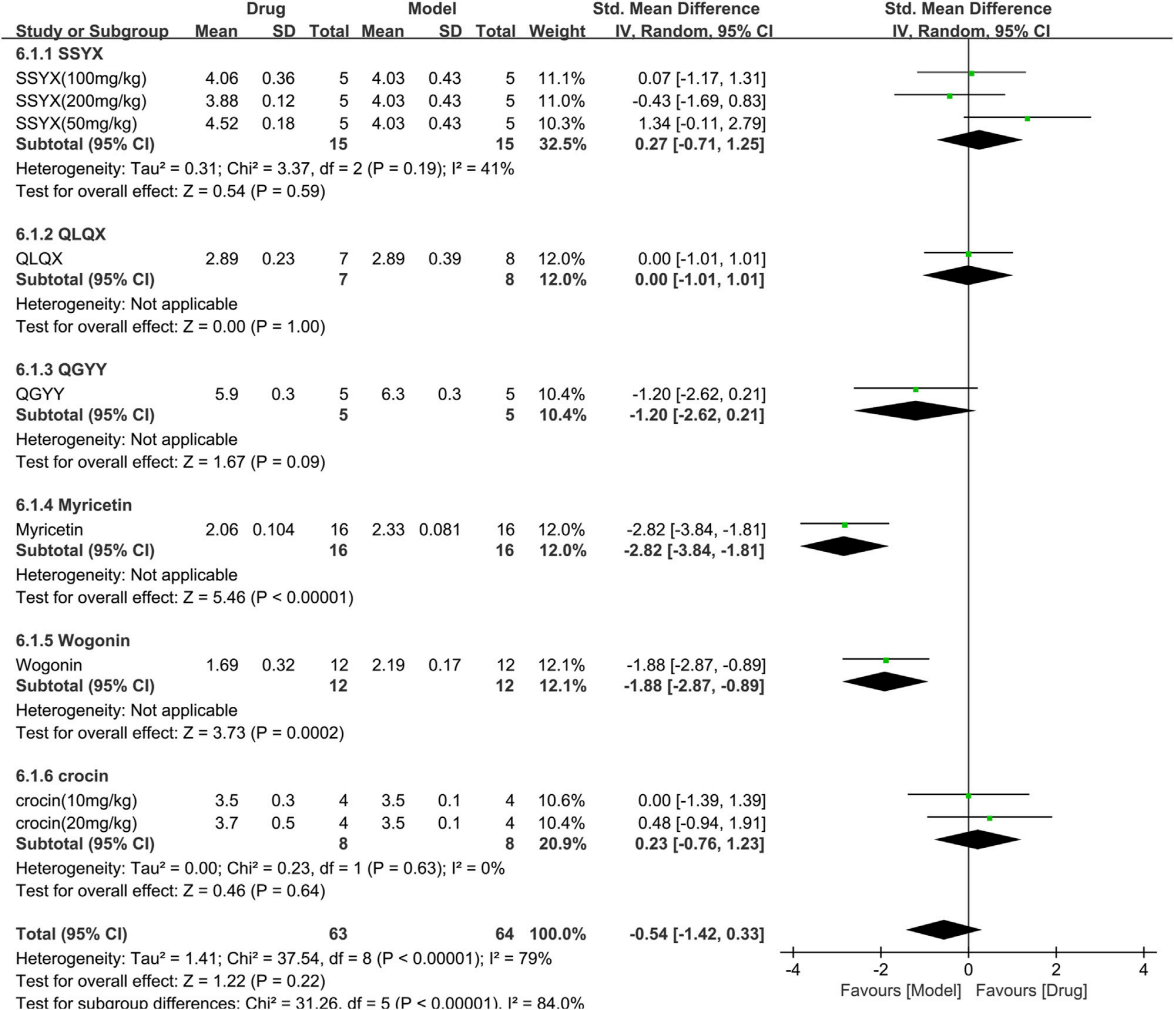
Effects of TCM on left ventricular internal dimension in systole (mm). Forest plots of the effect size were calculated using SMDs. The horizontal error bars represent the 95% confidence interval of individual studies.

Six studies involving 127 animals (63 in the TCM group and 64 in the control group) measured the left ventricular internal dimension in systole (LVIDs) and were included in the meta-analysis (Figure 9). The pooled results demonstrated that TCM had no significant effect on LVIDs [SMD = −0.54 (−1.42, 0.33), *p* = 0.22; 9 comparisons] Moreover, there was significant heterogeneity among the studies (χ^2^ = 37.54; I^2^ = 79%; df = 8; *p* < 0.00001). Subgroup analysis showed significant correlations (*p* < 0.00001). Myricetin and wogonin significantly reduced LVIDs compared to controls (*p* < 0.001). The forest plot indicated that myricetin had the most significant effect on reducing LVIDs.

#### 3.3.7 Efficacy of TCM on ejection fraction

**FIGURE 10:**
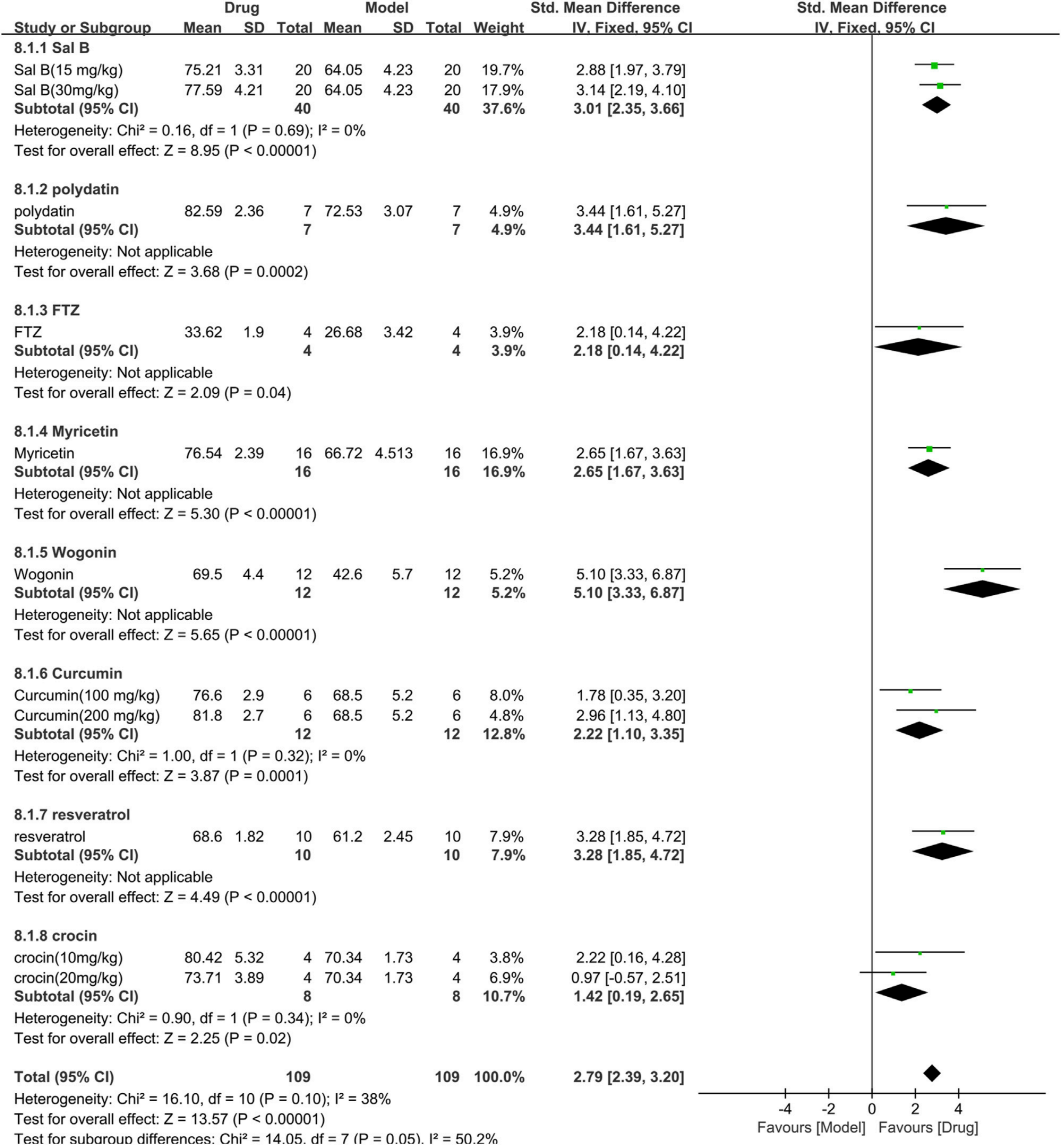
Effects of TCM on ejection fraction (%). Forest plots of the effect size were calculated using SMDs. The horizontal error bars represent the 95% confidence interval of individual studies.

For the ejection fraction, 8 studies (218 animals, 109 were in the TCM group and 109 were in the control group) were included in the meta-analysis (Figure 10). The aggregated results show that TCM significantly increased the ejection fraction [SMD = 2.79 (2.39,3.20), *p* < 0.00001; 11 comparisons], with moderate heterogeneity between studies (χ^2^ = 16.10; I^2^ = 38%; df = 10; *p* = 0.12). When analyzed with TCM as a subgroup, the difference was not statistically significant (*p* = 0.05), and all drugs increased the ejection fraction (*p* < 0.05). The forest plot indicated that wogonin had a more significant effect on increasing ejection fraction than other TCM drugs.

#### 3.3.8 Efficacy of TCM on fractional shortening

The FS was used in 8 studies (218 animals, 109 were in the TCM group and 109 were in the control group), which are included in the meta-analysis (Supplementary Figure S1). The meta-analysis showed that TCM significantly increased FS compared to control [SMD = 2.72 (2.32, 3.12), *p* < 0.00001; 11 comparisons), but with substantial heterogeneity among the studies (χ^2^ = 17.75; I^2^ = 44%; df = 10; *p* = 0.06). Subgroup analysis found no significant difference in FS improvement among different TCM drugs (*p* = 0.06). The forest plot indicated that polydatin had a more significant effect on improving FS compared to other TCM drugs.

#### 3.3.9 Efficacy of TCM on E/A

For the E/A 4 studies (117 animals, 58 were in the TCM group and 59 were in the control group) were included in the meta-analysis (Supplementary Figure S2). The meta-analysis revealed that TCM significantly improved E/A compared to control [SMD = 2.96 (2.41, 3.52; *p* < 0.00001; 5 comparisons) with moderate heterogeneity between studies (χ^2^ = 4.88; I^2^ = 18%; df = 4; *p* = 0.30). Subgroup analysis found no significant difference in E/A improvement among different TCM drugs (*p* = 0.20), and each drug significantly improved E/A compared to control (*p* < 0.05). The effect of QLQX on E/A was not significantly different from the control group (*p* = 0.07), while the effect of other TCM drugs on E/A was significantly better than control (*p* < 0.05). The forest plot indicated that polydatin had a more significant effect on improving E/A compared to other TCM drugs.

### 3.4 Publication bias

The study employed funnel plots (Supplementary Figure S3) and Egger’s test (Supplementary Figure S4) to identify potential publication bias. The results show that there was a significant publication bias related to body weight, HW/BW, blood glucose, triglyceride, and total cholesterol (*p* = 0.003, *p* < 0.001, *p* = 0.001, *p* < 0.001 and *p* = 0.006 respectively; Supplementary Figure S3A–E) However, no significant publication bias was found for ejection fraction and fractional shortening (*p* = 0.910 and *p* = 0.600, respectively; Supplementary Figure S3F–G). The study did not conduct a publication bias analysis on LVIDs and E/A indicators due to a limited number of included studies.

### 3.5 Sensitivity analysis

The study conducted sensitivity analyses by individually removing each study to assess its impact on the meta-analysis results. Supplementary Figure S5 shows all effect values falling within the 95% CI of the overall meta-analysis, indicating comparable effect sizes among studies. The results suggest the statistical validity and reliability of the findings.

### 3.6 Subgroup analysis

The results show high heterogeneity for BW, HW/BW, BG, TG, and TC. Therefore, subgroup analyses were performed to reveal the sources of heterogeneity, including variations in types of TCM, duration, modeling methods, and animal species.

#### 3.6.1 Effects of TCM types on efficacy

The study conducted subgroup analyses on BW, HW/BW, BG, TG, and TC and categorized TCM into monomers and compounds. Table 2 shows the resulting subgroup analysis. The monomer TCM group showed statistically significant improvements in BW, BG, and TG (*p* < 0.001), while the compound TCM group showed significant improvements in HW/BW and TC (*p* < 0.00001). Additionally, subgroup analysis indicated that heterogeneity of BW, HW/BW, BG, and TC was reduced in the compound group, while TG heterogeneity was reduced in the monomer group with statistical significance (*p* < 0.0001). The findings suggest that heterogeneity among studies might be attributed to differences in TCM types.

**TABLE 2:** Subgroup analysis of TCM types.

Outcomes	Monomers			Compounds		
	I^2^ (%)	*p*	SMD [95%CI]	I^2^ (%)	*p*	SMD [95%CI]
BW	78	<0.00001	1.83 [1.15,2.50]	54	0.002	1.29 [0.47,2.11]
HW/BW	79	<0.0001	−2.09 [−3.07,−1.12]	0	<0.00001	−2.37 [−2.80,−1.94]
BG	89	<0.00001	−2.37 [−3.41,−1.34]	61	<0.00001	−1.76 [−2.27,−1.24]
TG	54	<0.0001	−1.96 [−2.87,−1.04]	62	<0.00001	−1.86 [−2.50,−1.22]
TC	78	0.002	−1.38 [−2.24,−0.51]	54	<0.00001	−1.65 [−2.14,−1.16]

#### 3.6.2 Effects of duration on efficacy

The study conducted subgroup analyses for BW, HW/BW, BG, TG, and TC, dividing duration into two subgroups ≤56 days and >56 days. The subgroup analysis results are provided in Table 3. The TCM intervention duration of ≤56 days was significantly more effective in improving TG in DCM animals (*p* < 0.00001), whereas intervention duration >56 days was significantly better in enhancing BW, HW/BW, BG, and TC (*p* < 0.01) in DCM animals. However, subgroup analysis results for TC did not show any reduction in heterogeneity. The reduced heterogeneity observed in BW, HW/BW, BG, and TG suggests that duration may be a source of heterogeneity between studies; however, it is not considered a significant contributor to heterogeneity.

**TABLE 3:** Subgroup analysis of duration.

Outcomes	≤56			>56		
	I^2^ (%)	*p*	SMD [95%CI]	I^2^ (%)	*p*	SMD [95%CI]
BW	67	<0.00001	1.33 [0.78,1.89]	61	<0.00001	2.59 [1.70,3.49]
HW/BW	63	<0.00001	−2.15 [−2.69,−1.61]	51	0.0001	−2.86 [−4.31,−1.40]
BG	69	<0.00001	−1.30 [−1.78,−0.82]	85	<0.00001	−3.73 [−5.05,−2.41]
TG	64	<0.00001	−1.90 [−2.69,−1.11]	58	<0.00001	−1.88 [−2.61,−1.16]
TC	67	<0.00001	−1.36 [−1.85,−0.87]	81	0.002	−2.26 [−3.69,−0.83]

#### 3.6.3 Effects of modeling method on efficacy

Subgroup analysis was performed for BW, HW/BW, BG, TG, and TC. We divided the modeling method into three subgroups (STZ, HFD + STZ, and alloxan) for analysis. The results of subgroup analysis are presented in Table 4. Among the subgroups, treatment with a TCM exhibited a statistically significant improvement (*p* < 0.00001) in BW, HW/BW, and BG of HFD + STZ-induced DCM animals. However, subgroup analysis revealed no significant reduction in heterogeneity in BG and TC of HFD + STZ-induced DCM animals (*p* < 0.01). On the other hand, BW, HW/BW, and TG presented a reduction in heterogeneity. This reduction may be attributed to the modeling method.

**TABLE 4:** Subgroup analysis of the modeling method.

Outcomes	STZ			HFD + STZ			Alloxan		
	I^2^ (%)	*p*	SMD [95%CI]	I^2^ (%)	*p*	SMD [95%CI]	I^2^ (%)	*p*	SMD [95%CI]
BW	75	<0.00001	1.49 [0.91,2.08]	15	<0.00001	2.51 [1.67,3.35]	-	-	-
HW/BW	60	0.0002	−1.56 [−2.37,−0.75]	50	<0.00001	−2.52 [−3.08,−1.95]	-	-	-
BG	73	<0.0001	−1.48 [−2.20,−0.76]	85	<0.00001	−2.56 [−3.46,−1.67]	78	0.01	−1.82 [−3.25,−0.40]
TG	35	<0.00001	−2.60 [−3.64,−1.55]	48	<0.00001	−1.93 [−2.53,−1.33]	33	0.04	−0.78 [−1.51,−0.05]
TC	55	0.002	−1.60 [−2.63,−0.57]	76	<0.00001	−1.47 [−2.10,−0.84]	47	0.0001	−1.71 [−2.57,−0.85]

#### 3.6.4 Effects of animal species on efficacy

We performed subgroup analyses for BW, HW/BW, BG, TG, and TC. The animal species are divided into two subgroups (Mice and Rats). Subgroup analysis results are presented in Table 5. The effects of TCM on BW(*p* < 0.0001), TG (<0.00001), and TC(*p* < 0.01) were better in DCM mice than in DCM rats. The effects of TCM on HW/BW and BG were better in DCM rats than in DCM mice. Subgroup analysis showed heterogeneity of BW, HW/BW, BG, TG and, TC was reduced. The heterogeneity of BW, HW/BW, BG, TG, and TC may be caused by animal species.

**TABLE 5:** Subgroup analysis of animal species.

Outcomes	Mice			Rats		
	I^2^ (%)	*p*	SMD [95%CI]	I^2^ (%)	*p*	SMD [95%CI]
BW	82	<0.0001	1.98 [1.06,2.89]	62	<0.00001	1.41 [0.79,2.04]
HW/BW	0	<0.00001	−2.22 [-2.99,-1.45]	67	<0.00001	−2.23 [-2.83,-1.63]
BG	72	<0.0001	−1.60 [−2.34,−0.87]	84	<0.00001	−2.30 [−3.03,−1.56]
TG	68	<0.00001	−2.34 [−3.38,−1.30]	50	<0.00001	−1.61 [−2.18,−1.05]
TC	55	0.002	−1.60 [−2.63,−0.57]	73	<0.00001	−1.50 [−2.04,−0.96]

## 4 Discussion

### 4.1 Possible mechanisms of diabetic cardiomyopathy

The pathogenesis of diabetes encompasses various mechanisms that contribute to pancreatic beta-cell dysfunction and insulin resistance, including oxidative stress, endoplasmic reticulum stress, inflammation, autophagy, and DNA methylation (Ganesan et al., 2020). These mechanisms play crucial roles in the development of the disease. When exposed to elevated levels of glucose or fatty acids, the myocardium triggers the activation of NF-κB (Rahimi-Madiseh et al., 2017), a protein responsible for regulating immune responses and inflammation. NF-κB promotes the formation of NLRP3 inflammatory vesicles that release pro-inflammatory mediators (Asgary et al., 2014). Furthermore, heightened glucose levels disrupt mitochondrial function, leading to the generation of ROS (Hu et al., 2022). These ROS cause the aggregation of TXNIP with TRX (Feng et al., 2022), which then bind to NLRP3, initiating the activation of the NLRP3 inflammasome. This inflammasome activates caspase-1, which processes pro-inflammatory cytokines IL-1β and IL-18. ROS also stimulate NF-κB, thereby further facilitating the assembly of the NLRP3 inflammasome and the cleavage of caspase-1 (Egger et al., 1997; Engels et al., 2000). Consequently, this cascade results in the release of IL-1β and IL-18, potent inflammatory molecules implicated in the pathogenesis of diabetes. In diabetes, activated endothelial cells contribute to the early development of myocardial stiffness and diastolic dysfunction (Petersen and Shulman, 2018). They do so by promoting the uncoupling of endothelial NOS, resulting in the production of superoxide, hydrogen peroxide, and peroxynitrite. Consequently, the levels of NO are reduced (Zhou et al., 2010).

### 4.2 The efficacy of TCM

Based on this systematic review and meta-analysis, it is possible to infer that TCM has a significant impact on downregulating BG, TG, and TC in DCM animals, indicating its ability to modulate lipids and blood glucose. Moreover, the TCM treatment was highly effective in reducing myocardial hypertrophy and improving cardiac functions, as evidenced by the significant downregulation of HW/BW and the upregulation of EF, FS, and E/A with statistical significance.

### 4.3 Subgroup analysis

Table 2 presents the outcome measures, which are divided into two subgroups based on the type of traditional Chinese medicine (TCM): monomer and compound. Based on the results of subgroup analysis, it can be inferred that herbal monomers have a more pronounced impact on BG and TG levels in animals with DCM. Moreover, the treatment of DCM is closely linked to glucolipid metabolism (Montaigne et al., 2021). Thus, these findings imply that individual herbal monomers in TCM may offer greater advantages in enhancing glucose and lipid metaboism compared to TCM compounds.

Table 3 illustrates that even after categorizing the outcome measures into two subgroups based on their duration: ≤56 days and >56 days, a significant level of heterogeneity persisted across most subgroups. This implies that duration has a negligible impact on heterogeneity.

Table 4 showcases the outcome measures, which are categorized into three subgroups based on the animal modeling methods: STZ, HFD + STZ, and alloxan. While there was still some heterogeneity present in all subgroups, reductions were observed. Therefore, we can conclude that the primary source of heterogeneity in the included studies is the modeling approach, which aligns with previous research findings (Chen et al., 2023). The index related to lipid metabolism (TG) displayed significantly better results in the STZ group compared to the HFD + STZ group, potentially due to the impact of the high-fat diet on the measurement outcomes.

Table 5 demonstrates two subgroups, mice and rats, based on animal species. Results suggest that animal species is a contributing factor to heterogeneity, contradicting the outcomes of previous meta-analyses conducted on animal models of diabetes (Chen et al., 2022). Such disparity may stem from the inclusion of different animal strains within the study.

### 4.4 The possible mechanisms of TCM

#### 4.4.1 Anti-inflammatory action and decrease insulin resistance

The efficacy of wogonin, a herbal medicine traditionally used in mainland China and other Far East regions (Lee et al., 2015), was found to be significant in regulating body weight and EF, as indicated in the forest plot (Figures 4, 10). Wogonin can downregulate ROS-mediated NF-κB activation (Polier et al., 2011) and reduce hyperglycemia by increasing the transport of phosphorylated Akt and Glut4 to the plasma membrane (PM) in cardiac and skeletal muscle cells. Furthermore, treatment with wogonin resulted in a significant increase in Glut4 expression (Khan and Kamal, 2019). The hypoglycemic efficacy of mangiferin was the most significant among the 24 TCMs included (Figure 5). Previous studies have shown that long-term and short-term mangiferin treatments enhance PDH activity. This is partly due to the reduction of p-PDH levels caused by the downregulation of PDK4 by mangiferin (Apontes et al., 2014).

#### 4.4.2 Antioxidation of TCM

Oxidative stress damage is widely regarded as one of the important pathogenesis of diabetes. Mangiferin, taxifolin, polydatin, Nar, DOE, QGYY, Myricetin and Wogonin they all have significant antioxidant effects in different pathway, One study reported Myricetin attenuated oxidative stress through the acti-vation of the Nrf-2/ARE signal pathway (Liao et al., 2017). taxifolin and polydatin mediate through its inhibition of NADPH oxidase (Sun et al., 2014; Tan et al., 2020a). Nar can reduce the accumulation of unfolded or misfolded proteins in ER by increasing antioxidant enzyme activity and reducing lipid peroxide production, inhibiting ERS and reducing the expression of apoptosis related factors (Zhang et al., 2018). Although the antioxidant mechanism of each medicine is not necessarily the same, there is no doubt that they all have significant antioxidant effects.

#### 4.4.3 Efficacy in cardiac function of TCM

The forest plot analysis demonstrated that polydatin exhibited the exceptional efficacy in cardiac function, specifically in FS and E/A (Supplementary Figure S1, S2). Polydatin, derived from thuja’s rhizome and roots (Zhang et al., 2023), is a natural compound with a history of traditional use in treating inflammation, infection, jaundice, skin burns, and hyperlipidemia (Luo et al., 2022). Polydatin stimulates antifibrotic mechanisms and Nrf2-associated signaling pathways, while inhibiting NF-κB improves glycolipid metabolism following its administration (Karami et al., 2022). FTZ can decrease the interventricular septal thickness (Wang et al., 2021). YQHX, QLQX, Myricetin, Curcumin, resveratrol, crocin, can inhibit the apoptosis of cardiomyocytes. Moreover, the TCM treatment was highly effective in reducing myocardial hypertrophy and improving cardiac functions, as evidenced by the significant downregulation of HW/BW and the upregulation of EF, FS, and E/A with statistical significance. Whether the mechanism of TCM to reduce the effects of cell apoptosis and affect more modules.

#### 4.4.4 Other mechanism of TCM

Both hyperglycemia and hyperlipidemia are high-risk factors for diabetic cardiomyopathy. TCM has demonstrated the ability to regulate metabolism, including blood lipid and blood glucose levels. Our included studies have consistently reported the improvement of lipid levels in diabetic animals through TCM intervention, a result also supported by our meta-analysis with *p*-values less than 0.05 for all differences in Figure 7 and Figure 8. Based on this evidence, we conclude that TCM can effectively improve the disorder of blood lipid metabolism. Furthermore, our findings also indicate that TCM can improve the disorder of blood glucose metabolism, with *p*-values less than 0.05 for all differences in Figure 6. Moreover, a study (Diao et al., 2019) suggests that resveratrol promotes mitochondrial biosynthesis and inhibits excessive mitochondrial division in DCM, providing additional support for the benefits of TCM in this context.

### 4.5 Limitation

Most early studies were superficial and primarily concentrated on blood glucose and lipids, with few studies reported on diabetic cardiomyopathy and its mechanisms. The limited literature on diabetic cardiomyopathy could potentially compromise the study’s objectivity. Secondly, the quality of the literature acquired for the study was suboptimal, particularly with respect to randomization and blinded implementation. While most studies used the term “randomization,” they did not specifically detail the randomization method used. Additionally, none of the studies mentioned the application of blinded methods in the methods section. Thirdly, the high heterogeneity observed in the forest plots is a regular issue in meta-analyses of animal research, partly due to substandard study quality. Additionally, it could stem from various experimental factors, for example, the types of TCM, modeling methods, duration, and animal species.

## 5 Conclusion

Our study outcomes indicate that Traditional Chinese Medicine significantly improves heart function and controls the levels of blood glucose in animals with DCM, making it a viable treatment option. However, our study had several limitations due to the insufficient number of included studies, poor methodological quality, and a limited number of study animals. We acknowledge that our analysis was based on animal studies, which restricts generalization to humans. Therefore, further high-quality preclinical trials and clinical studies are required to validate our findings before fully applying TCM in the clinical setting.

## Data Availability

The original contributions presented in the study are included in the article/Supplementary Material, further inquiries can be directed to the corresponding author.

## References

[B1] Apontes P. Liu Z. Su K. Benard O. Youn D. Y. Li X. (2014). Mangiferin stimulates carbohydrate oxidation and protects against metabolic disorders induced by high-fat diets. Diabetes 63 (11), 3626–3636. 10.2337/db14-0006 24848064 PMC4207399

[B2] Asgary S. Rafieian-Kopaei M. Shamsi F. Najafi S. Sahebkar A. (2014). Biochemical and histopathological study of the anti-hyperglycemic and anti-hyperlipidemic effects of cornelian cherry (Cornus mas L.) in alloxan-induced diabetic rats. J. Complement. Integr. Med. 11 (2), 63–69. 10.1515/jcim-2013-0022 24710636

[B3] Chang W. Zhang M. Meng Z. Yu Y. Yao F. Hatch G. M. (2015). Berberine treatment prevents cardiac dysfunction and remodeling through activation of 5'-adenosine monophosphate-activated protein kinase in type 2 diabetic rats and in palmitate-induced hypertrophic H9c2 cells. Eur. J. Pharmacol. 769, 55–63. 10.1016/j.ejphar.2015.10.043 26522928

[B4] Chen R. Ma K. Li S. Zhou X. Chen H. (2023). Protective effects and mechanisms of opuntia polysaccharide in animal models of diabetes mellitus: A systematic review and meta-analysis. J. Ethnopharmacol. 312, 116490. 10.1016/j.jep.2023.116490 37054824

[B5] Chen X. M. Yang W. Q. Wang X. Chen C. Qian Z. M. Wang S. M. (2022). Effects of natural dihydrochalcones in sweet tea (lithocarpus polystachyus) on diabetes: A systematical review and meta-analysis of animal studies. Food Funct. 13 (11), 5899–5913. 10.1039/d2fo00245k 35583219

[B6] Couchie D. Vaisman B. Abderrazak A. Mahmood D. F. D. Hamza M. M. Canesi F. (2017). Human plasma thioredoxin-80 increases with age and in ApoE(-/-) mice induces inflammation, angiogenesis, and atherosclerosis. Circulation 136 (5), 464–475. 10.1161/circulationaha.117.027612 28473446 PMC8369893

[B7] Diao J. Wei J. Yan R. Fan G. Lin L. Chen M. (2019). Effects of resveratrol on regulation on UCP2 and cardiac function in diabetic rats. J. Physiol. Biochem. 75 (1), 39–51. 10.1007/s13105-018-0648-7 30225723

[B8] Dillmann W. H. (2019). Diabetic cardiomyopathy. Circ. Res. 124 (8), 1160–1162. 10.1161/circresaha.118.314665 30973809 PMC6578576

[B9] Dong Y. Zhao Q. Gao L. Wang W. (2021). Molecular mechanism of Astragalus-Angelicae drug pair in the treatment of diabetic cardiomyopathy. Chin. J. Exp. Formulary 27 (18), 16–24. 10.13422/j.cnki.syfjx.20211118

[B10] Egger M. Smith G. D. Phillips A. N. (1997). Meta-analysis: principles and procedures. Bmj 315 (7121), 1533–1537. 10.1136/bmj.315.7121.1533 9432252 PMC2127925

[B11] Engels E. A. Schmid C. H. Terrin N. Olkin I. Lau J. (2000). Heterogeneity and statistical significance in meta-analysis: an empirical study of 125 meta-analyses. Stat. Med. 19 (13), 1707–1728. 10.1002/1097-0258(20000715)19:13<1707:aid-sim491>3.0.co;2-p 10861773

[B12] Eriksson L. Nyström T. (2015). Antidiabetic agents and endothelial dysfunction - beyond glucose control. Basic Clin. Pharmacol. Toxicol. 117 (1), 15–25. 10.1111/bcpt.12402 25827165

[B13] Ernande L. Derumeaux G. (2012). Diabetic cardiomyopathy: myth or reality? Arch. Cardiovasc Dis. 105 (4), 218–225. 10.1016/j.acvd.2011.11.007 22633296

[B14] Evangelista I. Nuti R. Picchioni T. Dotta F. Palazzuoli A. (2019). Molecular dysfunction and phenotypic derangement in diabetic cardiomyopathy. Int. J. Mol. Sci. 20 (13), 3264. 10.3390/ijms20133264 31269778 PMC6651260

[B15] Feidantsis K. Mellidis K. Galatou E. Sinakos Z. Lazou A. (2018). Treatment with crocin improves cardiac dysfunction by normalizing autophagy and inhibiting apoptosis in STZ-induced diabetic cardiomyopathy. Nutr. Metab. Cardiovasc Dis. 28 (9), 952–961. 10.1016/j.numecd.2018.06.005 30017436

[B16] Feng H. Wu T. Zhou Q. Li H. Liu T. Ma X. (2022). Protective effect and possible mechanisms of artemisinin and its derivatives for diabetic nephropathy: A systematic review and meta-analysis in animal models. Oxid. Med. Cell Longev. 2022, 5401760. 10.1155/2022/5401760 35528521 PMC9073547

[B17] Franssen C. Chen S. Unger A. Korkmaz H. I. De Keulenaer G. W. Tschöpe C. (2016). Myocardial microvascular inflammatory endothelial activation in heart failure with preserved ejection fraction. JACC Heart Fail 4 (4), 312–324. 10.1016/j.jchf.2015.10.007 26682792

[B18] Ganesan D. Albert A. Paul E. Ananthapadmanabhan K. Andiappan R. Sadasivam S. G. (2020). Rutin ameliorates metabolic acidosis and fibrosis in alloxan induced diabetic nephropathy and cardiomyopathy in experimental rats. Mol. Cell Biochem. 471 (1-2), 41–50. 10.1007/s11010-020-03758-y 32529498

[B19] Guo R. Nair S. (2017). Role of microRNA in diabetic cardiomyopathy: from mechanism to intervention. Biochim. Biophys. Acta Mol. Basis Dis. 1863 (8), 2070–2077. 10.1016/j.bbadis.2017.03.013 28344129 PMC5475364

[B20] Guo X. Xue M. Li C. J. Yang W. Wang S. S. Ma Z. J. (2016). Protective effects of triptolide on TLR4 mediated autoimmune and inflammatory response induced myocardial fibrosis in diabetic cardiomyopathy. J. Ethnopharmacol. 193, 333–344. 10.1016/j.jep.2016.08.029 27558948

[B21] He F. Huang Y. Song Z. Zhou H. J. Zhang H. Perry R. J. (2021). Mitophagy-mediated adipose inflammation contributes to type 2 diabetes with hepatic insulin resistance. J. Exp. Med. 218 (3), e20201416. 10.1084/jem.20201416 33315085 PMC7927432

[B22] Hou J. Zheng D. Fung G. Deng H. Chen L. Liang J. (2016). Mangiferin suppressed advanced glycation end products (AGEs) through NF-κB deactivation and displayed anti-inflammatory effects in streptozotocin and high fat diet-diabetic cardiomyopathy rats. Can. J. Physiol. Pharmacol. 94 (3), 332–340. 10.1139/cjpp-2015-0073 26751764

[B23] Hu T. Yue J. Tang Q. Cheng K. W. Chen F. Peng M. (2022). The effect of quercetin on diabetic nephropathy (DN): A systematic review and meta-analysis of animal studies. Food Funct. 13 (9), 4789–4803. 10.1039/d1fo03958j 35416188

[B24] Jankauskas S. S. Kansakar U. Varzideh F. Wilson S. Mone P. Lombardi A. (2021). Heart failure in diabetes. Metabolism 125, 154910. 10.1016/j.metabol.2021.154910 34627874 PMC8941799

[B25] Karami A. Fakhri S. Kooshki L. Khan H. (2022). Polydatin: pharmacological mechanisms, therapeutic targets, biological activities, and health benefits. Molecules 27 (19), 6474. 10.3390/molecules27196474 36235012 PMC9572446

[B26] Khan S. Kamal M. A. (2019). Wogonin alleviates hyperglycemia through increased glucose entry into cells via AKT/GLUT4 pathway. Curr. Pharm. Des. 25 (23), 2602–2606. 10.2174/1381612825666190722115410 31333118

[B27] Khan S. Zhang D. Zhang Y. Li M. Wang C. (2016). Wogonin attenuates diabetic cardiomyopathy through its anti-inflammatory and anti-oxidative properties. Mol. Cell Endocrinol. 428, 101–108. 10.1016/j.mce.2016.03.025 27013352

[B28] Lawrence T. (2009). The nuclear factor NF-kappaB pathway in inflammation. Cold Spring Harb. Perspect. Biol. 1 (6), a001651. 10.1101/cshperspect.a001651 20457564 PMC2882124

[B29] Lee W. Ku S. K. Bae J. S. (2015). Anti-inflammatory effects of baicalin, baicalein, and wogonin *in vitro* and *in vivo* . Inflammation 38 (1), 110–125. 10.1007/s10753-014-0013-0 25249339

[B30] Lehrke M. Marx N. (2017). Diabetes mellitus and heart failure. Am. J. Med. 130 (6), S40–s50. 10.1016/j.amjmed.2017.04.010 28526183

[B31] Li C. L. Liu B. Wang Z. Y. Xie F. Qiao W. Cheng J. (2020). Salvianolic acid B improves myocardial function in diabetic cardiomyopathy by suppressing IGFBP3. J. Mol. Cell Cardiol. 139, 98–112. 10.1016/j.yjmcc.2020.01.009 31982427

[B32] Liao H. H. Zhu J. X. Feng H. Ni J. Zhang N. Chen S. (2017). Myricetin possesses potential protective effects on diabetic cardiomyopathy through inhibiting iκbα/nfκb and enhancing Nrf2/HO-1. Oxid. Med. Cell Longev. 2017, 8370593. 10.1155/2017/8370593 29147465 PMC5632894

[B33] Loffroy R. Bernard S. Sérusclat A. Boussel L. Bonnefoy E. D'Athis P. (2009). Noninvasive assessment of the prevalence and characteristics of coronary atherosclerotic plaques by multidetector computed tomography in asymptomatic type 2 diabetic patients at high risk of significant coronary artery disease: A preliminary study. Arch. Cardiovasc Dis. 102 (8-9), 607–615. 10.1016/j.acvd.2009.04.007 19786264

[B34] Lorenzo-Almorós A. Cepeda-Rodrigo J. M. Lorenzo Ó. (2022). Diabetic cardiomyopathy. Rev. Clin. Esp. Barc. 222 (2), 100–111. 10.1016/j.rceng.2019.10.012 35115137

[B35] Luo J. Chen S. Wang L. Zhao X. Piao C. (2022). Pharmacological effects of polydatin in the treatment of metabolic diseases: A review. Phytomedicine 102, 154161. 10.1016/j.phymed.2022.154161 35636169

[B36] Meng T. Li X. Li C. Liu J. Chang H. Jiang N. (2022). Natural products of traditional Chinese medicine treat atherosclerosis by regulating inflammatory and oxidative stress pathways. Front. Pharmacol. 13, 997598. 10.3389/fphar.2022.997598 36249778 PMC9563010

[B37] Montaigne D. Butruille L. Staels B. (2021). PPAR control of metabolism and cardiovascular functions. Nat. Rev. Cardiol. 18 (12), 809–823. 10.1038/s41569-021-00569-6 34127848

[B38] Nakamura K. Miyoshi T. Yoshida M. Akagi S. Saito Y. Ejiri K. (2022). Pathophysiology and treatment of diabetic cardiomyopathy and heart failure in patients with diabetes mellitus. Int. J. Mol. Sci. 23 (7), 3587. 10.3390/ijms23073587 35408946 PMC8999085

[B39] Page M. J. McKenzie J. E. Bossuyt P. M. Boutron I. Hoffmann T. C. Mulrow C. D. (2021). The PRISMA 2020 statement: an updated guideline for reporting systematic reviews. Bmj 372, n71. 10.1136/bmj.n71 33782057 PMC8005924

[B40] Paolillo S. Marsico F. Prastaro M. Renga F. Esposito L. De Martino F. (2019). Diabetic cardiomyopathy: definition, diagnosis, and therapeutic implications. Heart Fail Clin. 15 (3), 341–347. 10.1016/j.hfc.2019.02.003 31079692

[B41] Petersen M. C. Shulman G. I. (2018). Mechanisms of insulin action and insulin resistance. Physiol. Rev. 98 (4), 2133–2223. 10.1152/physrev.00063.2017 30067154 PMC6170977

[B42] Polier G. Ding J. Konkimalla B. V. Eick D. Ribeiro N. Köhler R. (2011). Wogonin and related natural flavones are inhibitors of CDK9 that induce apoptosis in cancer cells by transcriptional suppression of Mcl-1. Cell Death Dis. 2 (7), e182. 10.1038/cddis.2011.66 21776020 PMC3199715

[B43] Rahimi-Madiseh M. Heidarian E. Kheiri S. Rafieian-Kopaei M. (2017). Effect of hydroalcoholic Allium ampeloprasum extract on oxidative stress, diabetes mellitus and dyslipidemia in alloxan-induced diabetic rats. Biomed. Pharmacother. 86, 363–367. 10.1016/j.biopha.2016.12.028 28011384

[B44] Scirica B. M. Braunwald E. Raz I. Cavender M. A. Morrow D. A. Jarolim P. (2015). Heart failure, saxagliptin, and diabetes mellitus: observations from the SAVOR-TIMI 53 randomized trial. Circulation 132 (15), e198. 10.1161/cir.0000000000000330 26459088

[B45] Seferović P. M. Paulus W. J. (2015). Clinical diabetic cardiomyopathy: A two-faced disease with restrictive and dilated phenotypes. Eur. Heart J. 36 (27), 1718–1727. 10.1093/eurheartj/ehv134 25888006

[B46] Seferovic P. M. Petrie M. C. Filippatos G. S. Anker S. D. Rosano G. Bauersachs J. (2018). Type 2 diabetes mellitus and heart failure: A position statement from the heart failure association of the European society of cardiology. Eur. J. Heart Fail 20 (5), 853–872. 10.1002/ejhf.1170 29520964

[B47] Shao B. Z. Xu Z. Q. Han B. Z. Su D. F. Liu C. (2015). NLRP3 inflammasome and its inhibitors: A review. Front. Pharmacol. 6, 262. 10.3389/fphar.2015.00262 26594174 PMC4633676

[B48] Shen N. Li X. Zhou T. Bilal M. U. Du N. Hu Y. (2014). Shensong Yangxin Capsule prevents diabetic myocardial fibrosis by inhibiting TGF-β1/Smad signaling. J. Ethnopharmacol. 157, 161–170. 10.1016/j.jep.2014.09.035 25267579

[B49] Shi H. Wang L. Fang Z. H. Ni Y. Q. Shen A. L. Liu P. P. (2019). Experimental study on effect and mechanism of Danzhi Jiangtang Capsules on diabetic myocardial injury. Zhongguo Zhong Yao Za Zhi 44 (23), 5159–5165. 10.19540/j.cnki.cjcmm.20191015.401 32237353

[B50] Sun X. Chen R. C. Yang Z. H. Sun G. B. Wang M. Ma X. J. (2014). Taxifolin prevents diabetic cardiomyopathy *in vivo* and *in vitro* by inhibition of oxidative stress and cell apoptosis. Food Chem. Toxicol. 63, 221–232. 10.1016/j.fct.2013.11.013 24269735

[B51] Tan Y. Zhang Z. Zheng C. Wintergerst K. A. Keller B. B. Cai L. (2020b). Mechanisms of diabetic cardiomyopathy and potential therapeutic strategies: preclinical and clinical evidence. Nat. Rev. Cardiol. 17 (9), 585–607. 10.1038/s41569-020-0339-2 32080423 PMC7849055

[B52] Tan Y. Y. Chen L. X. Fang L. Zhang Q. (2020a). Cardioprotective effects of polydatin against myocardial injury in diabetic rats via inhibition of NADPH oxidase and NF-κB activities. BMC Complement. Med. Ther. 20 (1), 378. 10.1186/s12906-020-03177-y 33308195 PMC7733248

[B53] Wang J. Ma Q. Li Y. Li P. Wang M. Wang T. (2020). Research progress on Traditional Chinese Medicine syndromes of diabetes mellitus. Biomed. Pharmacother. 121, 109565. 10.1016/j.biopha.2019.109565 31704615

[B54] Wang L. Wu H. Deng Y. Zhang S. Wei Q. Yang Q. (2021). FTZ ameliorates diabetic cardiomyopathy by inhibiting inflammation and cardiac fibrosis in the streptozotocin-induced model. Evid. Based Complement. Altern. Med. 2021, 5582567. 10.1155/2021/5582567 PMC8492284 34621323

[B55] Wang X. Huang J. Wang S. Ni Q. (2018). The Chinese herb yi-qi-huo-xue protects cardiomyocyte function in diabetic cardiomyopathy. Evid. Based Complement. Altern. Med. 2018, 7316840. 10.1155/2018/7316840 PMC5960524 29853969

[B56] Wei Q. Zhu T. Xiao X. Sun L. Zhang Z. Huang T. (2019). Dioscin attenuates myocardial damages in diabetic rats maybe by regulating NO-sGC-cGMP-PKG pathway. Ann. Clin. Lab. Sci. 49 (1), 97–104. 30814084

[B57] Wu X. Zhang T. Lyu P. Chen M. Ni G. Cheng H. (2021). Traditional Chinese medication qiliqiangxin attenuates diabetic cardiomyopathy via activating PPARγ. Front. Cardiovasc Med. 8, 698056. 10.3389/fcvm.2021.698056 34336956 PMC8322738

[B58] Xiao E. Luo L. (2018). Alternative therapies for diabetes: A comparison of western and traditional Chinese medicine (TCM) approaches. Curr. Diabetes Rev. 14 (6), 487–496. 10.2174/1573399813666170519103230 28523995

[B59] Yao R. Cao Y. Wang C. Xu L. Zhang X. Deng Y. (2021). Taohuajing reduces oxidative stress and inflammation in diabetic cardiomyopathy through the sirtuin 1/nucleotide-binding oligomerization domain-like receptor protein 3 pathway. BMC Complement. Med. Ther. 21 (1), 78. 10.1186/s12906-021-03218-0 33637069 PMC7913206

[B60] Yu W. Wu J. Cai F. Xiang J. Zha W. Fan D. (2012). Curcumin alleviates diabetic cardiomyopathy in experimental diabetic rats. PLoS One 7 (12), e52013. 10.1371/journal.pone.0052013 23251674 PMC3522633

[B61] Zhang Y. F. Meng N. N. Li H. Z. Wen Y. J. Liu J. T. Zhang C. L. (2018). Effect of naringin on oxidative stress and endoplasmic reticulum stress in diabetic cardiomyopathy. Zhongguo Zhong Yao Za Zhi 43 (3), 596–602. 10.19540/j.cnki.cjcmm.2018.0013 29600628

[B62] Zhang Z. Sun Z. Jia R. Jiang D. Xu Z. Zhang Y. (2023). Protective effects of polydatin against bone and joint disorders: the *in vitro* and *in vivo* evidence so far. Nutr. Res. Rev., 1–12. 10.1017/s0954422423000082 37088535

[B63] Zhang Z. Zhang D. Dou M. Li Z. Zhang J. Zhao X. (2016). Dendrobium officinale Kimura et Migo attenuates diabetic cardiomyopathy through inhibiting oxidative stress, inflammation and fibrosis in streptozotocin-induced mice. Biomed. Pharmacother. 84, 1350–1358. 10.1016/j.biopha.2016.10.074 27802903

[B64] Zhou R. Tardivel A. Thorens B. Choi I. Tschopp J. (2010). Thioredoxin-interacting protein links oxidative stress to inflammasome activation. Nat. Immunol. 11 (2), 136–140. 10.1038/ni.1831 20023662

[B65] Zhou R. Yazdi A. S. Menu P. Tschopp J. (2011). A role for mitochondria in NLRP3 inflammasome activation. Nature 469 (7329), 221–225. 10.1038/nature09663 21124315

