# Supplementary material for: Efficacy of traditional Chinese medicine on diabetic cardiomyopathy in animal models: a systematic review and meta-analysis
Source: Front Pharmacol. 2023 Sep 28;14:1253572. doi: 10.3389/fphar.2023.1253572 (PMC10578493; doi:10.3389/fphar.2023.1253572)
Supplement: Supplementary file 1 [file DataSheet1.DOCX]

Supplementary Material

Efficacy of traditional Chinese medicine on diabetic cardiomyopathy in animal models: A systematic review and meta-analysis

Longxiao Hu^1,2†^, Longxin Qian^1†^, Aochuan Sun^1,2^, Guida Cai^4^, Yunxiao Gao^2^, Yue Yuan^2^, Xiaoxiao Chen^2^, Yunyao Jiang^3^, Jianxun Liu^2^, Junguo Ren^2*^

†These authors contributed equally to this work and share first authorship

^1^Beijing University of Chinese Medicine, Beijing, China

^2^Institute of Basic Medical Sciences, Xiyuan Hospital, China Academy of Chinese Medical Sciences, Beijing, China

^3^Institute for Chinese Materia Medica, School of Pharmaceutical Sciences, Tsinghua University, Beijing, China

^4^ Guangdong Pharmaceutical University, Guangdong, China

*** Correspondence:**Junguo Ren
reek2003@163.com

# Supplementary Figures and Tables

## Supplementary Figures


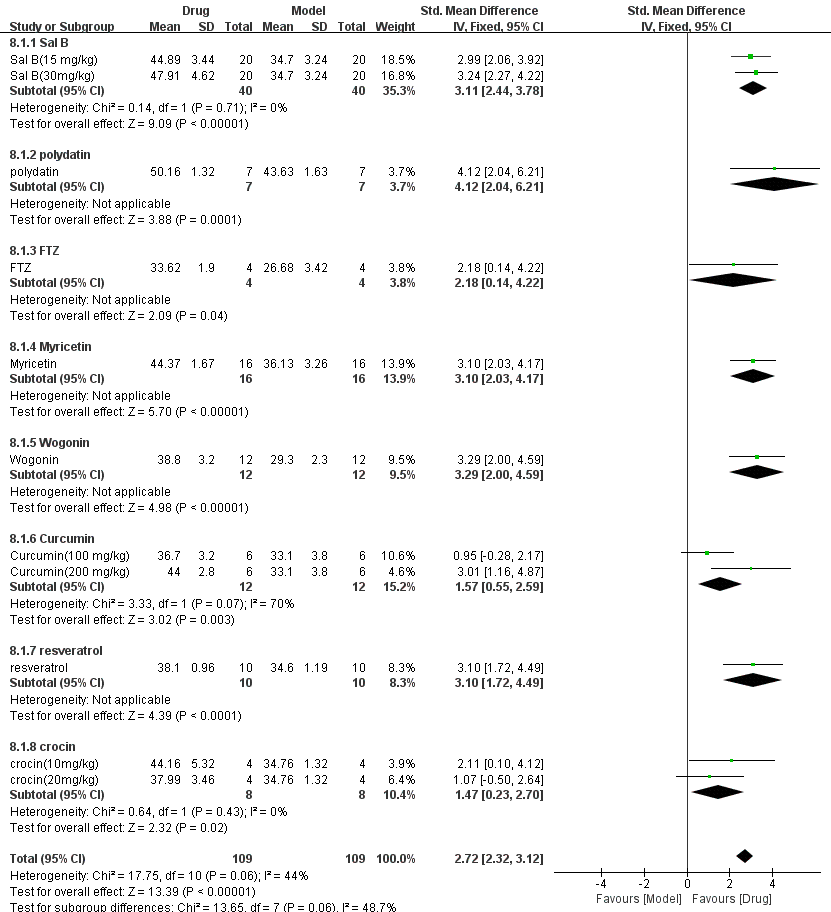


**Supplementary Figure S1.** Effects of TCM on fractional shortening(%). Forest plots of the effect size were calculated using SMDs. The horizontal error bars represent the 95% confidence interval of individual studies.


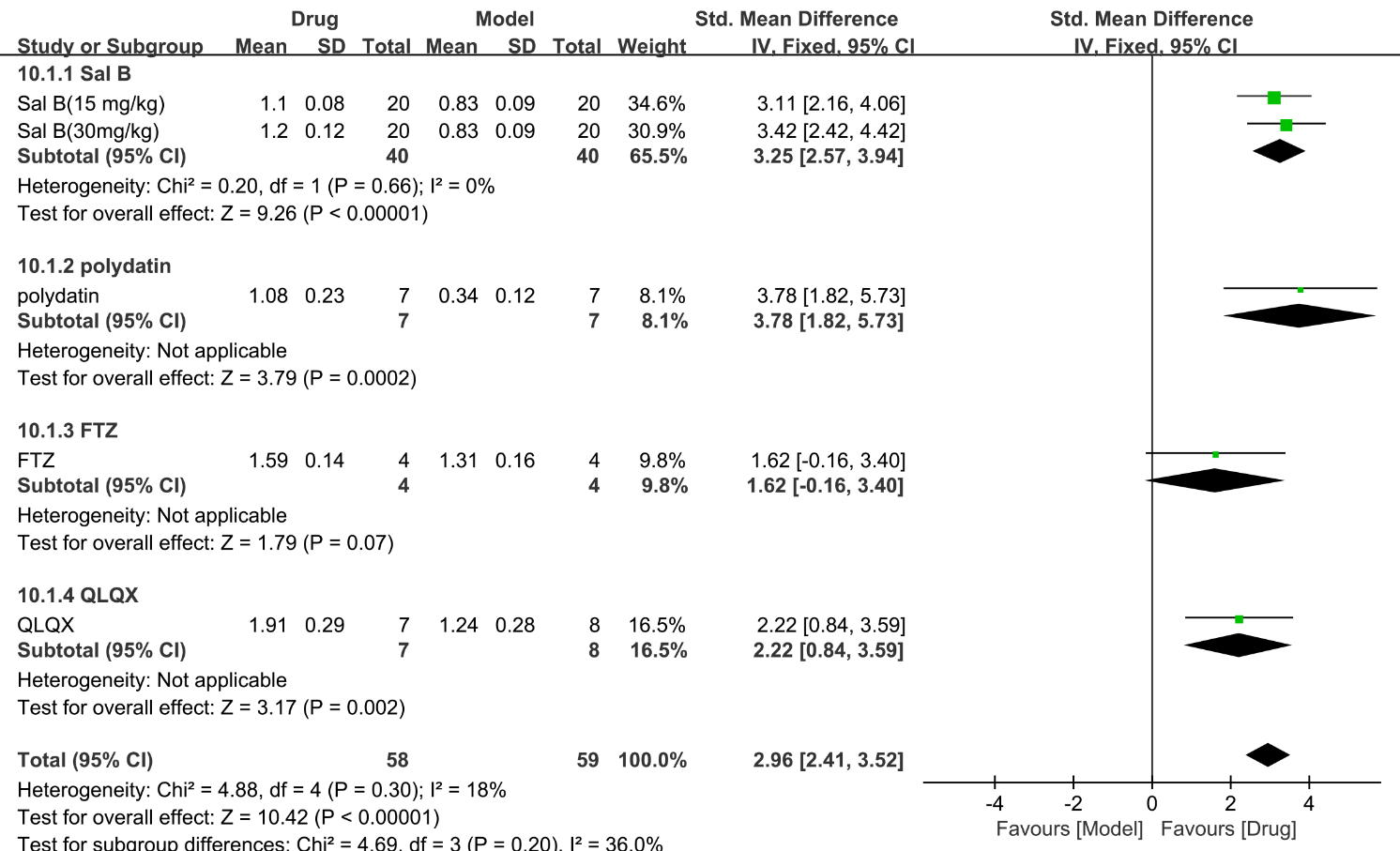


**Supplementary Figure S2.** Effects of TCM on E/A. Forest plots of the effect size were calculated using SMDs. The horizontal error bars represent the 95% confidence interval of individual studies.


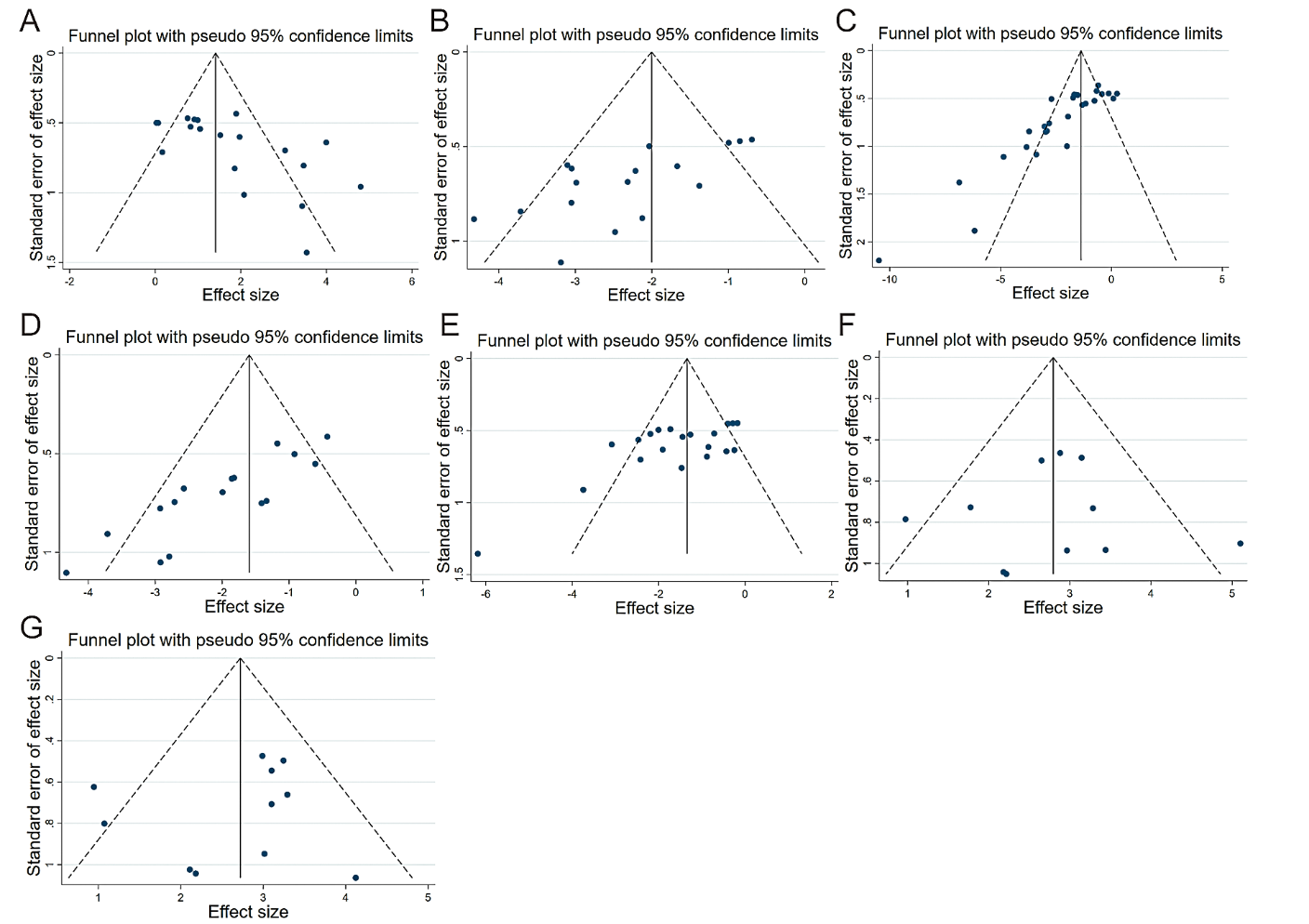


**Supplementary Figure S3.** Funnel plots for the body weight (A), HW/BW (B), blood glucose (C), triglyceride (D), total cholesterol (E), ejection fraction(F) and fractional shortening(G) showing the publication bias.


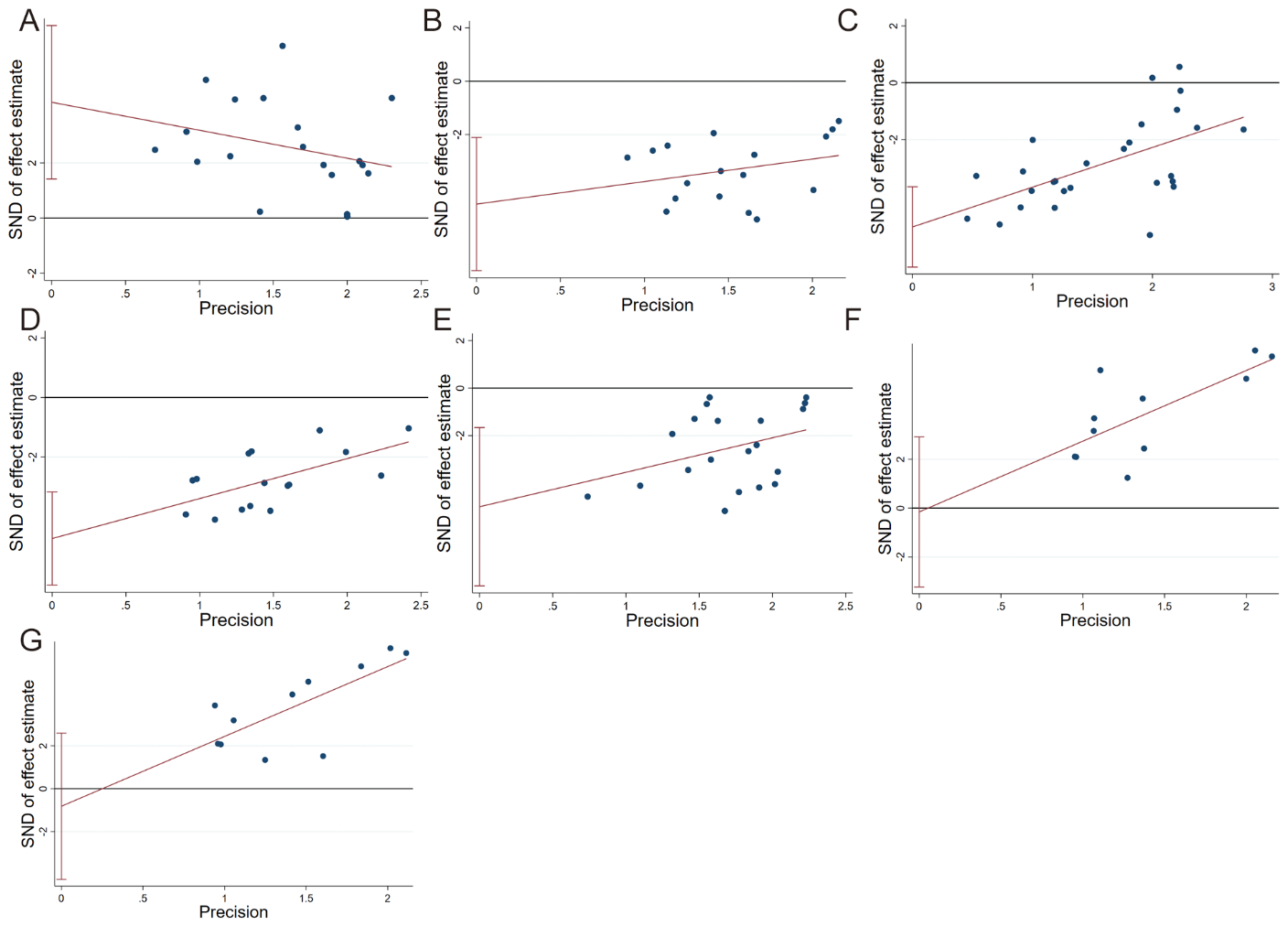
 **Supplementary Figure S4.** Egger’s regression for the body weight (A), HW/BW (B), blood glucose (C), triglyceride (D), total cholesterol (E), ejection fraction(F) and fractional shortening(G) showing the publication bias.
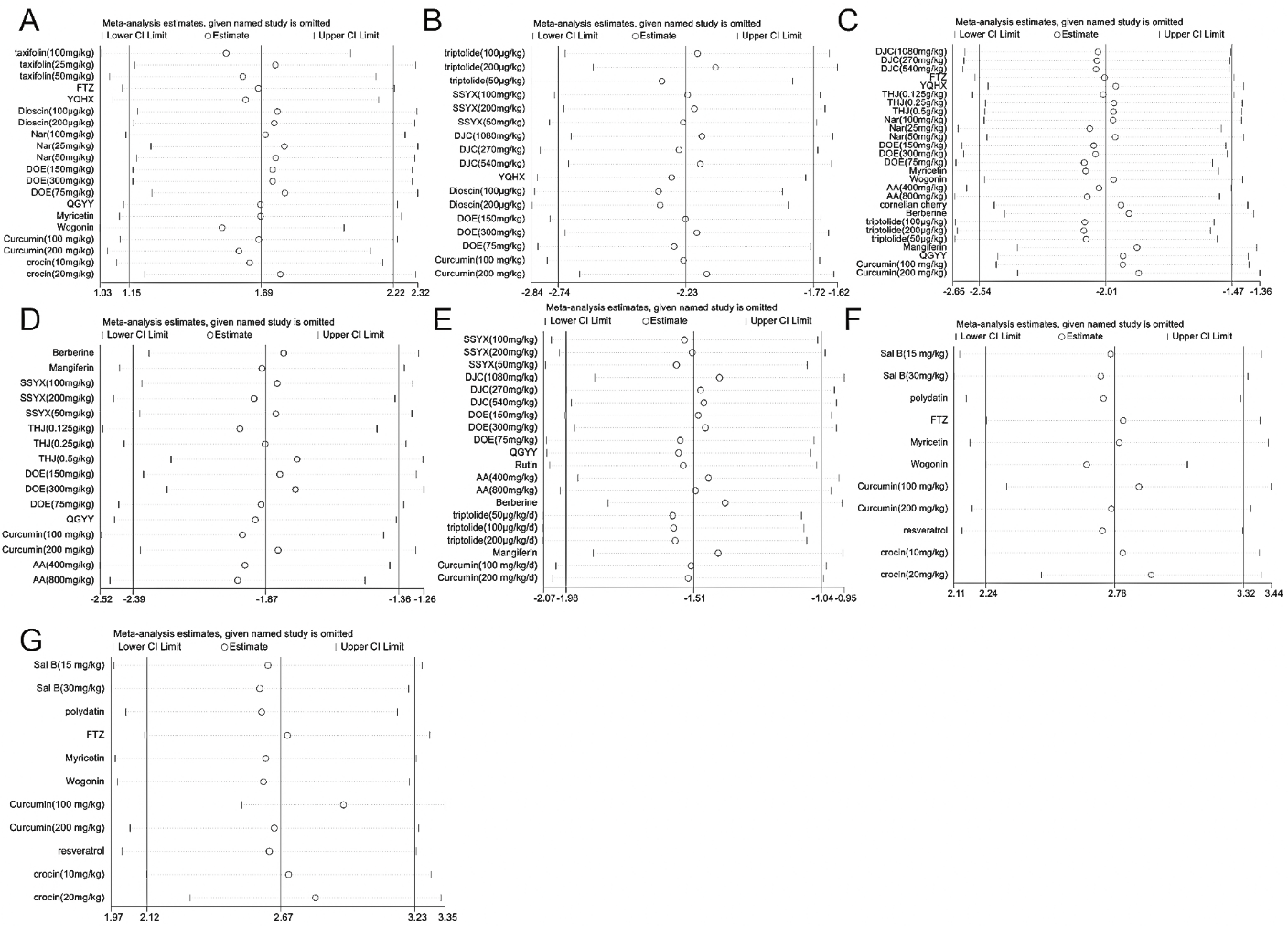
 **Supplementary Figure S5.** Sensitivity analysis for the body weight (A), HW/BW (B), blood glucose (C), triglyceride (D), total cholesterol (E), ejection fraction(F) and fractional shortening(G) showing the publication bias.
